# The combination of *Lonicerae Japonicae Flos* and *Forsythiae Fructus* herb-pair alleviated inflammation in liver fibrosis

**DOI:** 10.3389/fphar.2022.984611

**Published:** 2022-08-19

**Authors:** Jing-Bei Zhang, Hong-Liu Jin, Xiao-Ying Feng, Sen-ling Feng, Wen-Ting Zhu, Hong-Mei Nan, Zhong-Wen Yuan

**Affiliations:** ^1^ Collage of Chinese Medicine, Changchun University of Chinese Medicine, Jilin, China; ^2^ Department of Pharmacy, The Third Affiliated Hospital of Guangzhou Medical University, Guangzhou, China; ^3^ School of Pharmaceutical Sciences, Guangzhou Medical University, Guangzhou, China; ^4^ Guangdong Provincial Key Laboratory of Major Obstetric Diseases, Guangzhou Medical University, Guangzhou, China; ^5^ Department of Encephalopathy, Affiliated Hospital of Changchun University of Chinese Medicine, Jilin, China

**Keywords:** *Lonicerae Japonicae Flos*, *Forsythiae Fructus*, liver fibrosis, inflammation, oxidative stress, traditional Chinese medicine

## Abstract

**Objective:** To explore the active components and epigenetic regulation mechanism underlying the anti-inflammatory effects of *Lonicerae Japonicae Flos* and *Forsythiae Fructus* herb-pair (LFP) in carbon tetrachloride (CCl_4_)-induced rat liver fibrosis.

**Methods:** The main active ingredients and disease-related gene targets of LFP were determined using TCMSP and UniProt, and liver fibrosis disease targets were screened in the GeneCards database. A network was constructed with Cytoscape 3.8.0 and the STRING database, and potential protein functions were analyzed using bioinformatics analysis. Based on these analyses, we determined the main active ingredients of LFP and evaluated their effects in a CCl_4_-induced rat liver fibrosis model. Serum biochemical indices were measured using commercial kits, hepatocyte tissue damage and collagen deposition were evaluated by histopathological studies, and myofibroblast activation and inflammation were detected by reverse transcription-polymerase chain reaction (RT-PCR) and western blotting. High-performance liquid chromatography-mass spectrometry was performed to determine the levels of homocysteine, reduced glutathione, and oxidized glutathione, which are involved in inflammation and oxidative stress.

**Results:** The main active components of LFP were quercetin, kaempferol, and luteolin, and its main targets were α-smooth muscle actin, cyclooxygenase-2, formyl-peptide receptor-2, prostaglandin-endoperoxide synthase 1, nuclear receptor coactivator-2, interleukinβ, tumor necrosis factor α, CXC motif chemokine ligand 14, and transforming growth factor β1. A combination of quercetin, kaempferol, and luteolin alleviated the symptoms of liver fibrosis.

**Conclusion:** The results of this study support the role of LFP in the treatment of liver fibrosis, and reveal that LFP reduces collagen formation, inflammation, and oxidative stress. This study suggests a potential mechanism of action of LFP in the treatment of liver fibrosis.

## 1 Introduction

Liver fibrosis is a complex compensatory repair response caused by chronic liver damage due to a viral infection, autoimmune disorder, or drugs, and is characterized by excessive deposition of extracellular matrix, ultimately leading to cirrhosis with high morbidity and mortality (Cheng et al., 2019; Chen et al., 2021b; Liang et al., 2021).

The epigenetic regulation mechanism of liver fibrosis is intricate, mainly including DNA methylation, histone modification, microRNAs (miRNAs), etc., It regulates the occurrence and development of liver fibrosis by affecting the expression of fibrosis-related genes, the activation, proliferation, apoptosis of hepatic stellate cells (HSC) and myofibroblast (MF) differentiation. There is research (Komatsu et al., 2012) has shown that the methylation level of secreted pyrophosphoprotein 1 (Spp1), a gene involved in inflammation-induced liver fibrosis, is low and its expression is up-regulated. Inaddition, anti-fibrosis genes are often hypermethylated in liver fibrosis. Study (Pan et al., 2018) has shown that hypermethylation of cyclic prostaglandin synthase 1 (PTGS1) genes represses their transcription and ultimately upregulates the expression of α-SMA and COL1A1 genes, promoting HSC activation and liver fibrosis. Furthermore, acetylation and methylation are more common in liver fibrosis, and up-regulates gene expression or down-regulates particular gene expression.

Chinese herbs are commonly used in traditional Chinese medicine (TCM) for the treatment of liver disease. *Lonicerae Japonicae Flos*, a dried flower bud or flower of the honeysuckle family, exhibits heat-clearing and detoxifying effects. *Lonicerae Japonicae Flos* has been reported to have a protective effect against liver damage induced by various factors (Hu et al., 2020a). *Forsythiae Fructus*, a plant of the genus *L. forsythia*, exhibits several functions including wind-heat dispersion, heat clearance, detoxication, swelling reduction, and knot dissipation. *Forsythiae Fructus* can scavenge oxygen free radicals, inhibit hemolysis of red blood cells induced by hydrogen peroxide, and reduce the accumulation of the peroxidation product malondialdehyde, thereby inhibiting mitochondrial oxidative damage and exerting hepatoprotective effects (Yang et al., 2013; Chen et al., 2021b).

The combination of the two herbs was first used in an ancient TCM, “YinQiao Powder,” which has detoxifying, heat-clearing, and anti-inflammatory properties. It was recorded in the famous works “Wen Bing Tiao Bian” and is prescribed for the treatment of hepatopathy. However, the mechanism of action of this combination in the treatment of liver fibrosis remains unclear.

Due to the complex composition of TCM, its target pathways and mechanisms of action in the treatment of diseases are not clear; therefore, its scope of use is limited (Jiang et al., 2020). Network pharmacology is a scientific method for molecular target prediction using systems biology and network analysis. Network-based methods are used to visualize complex target disease networks, which are important for understanding the regulatory mechanisms of multi-component and multi-target TCM (Zhou et al., 2021).

In this study, we combined network pharmacology and pharmacological experiments. First, we identified the main active components and targets of *Lonicerae Japonicae Flos*-*Forsythiae Fructus* herb-pair (LFP) using bioinformatics and constructed a component-target network. Additionally, we performed functional enrichment analyses of target genes to explore the possible therapeutic mechanisms in the treatment of liver fibrosis. We further used a carbon tetrachloride (CCl_4_)-induced rat liver fibrosis model to elucidate the mechanisms underlying the effects of LFP and its protective effects on the liver by investigating fibroblast differentiation, inflammatory reactions, energy balance, lipid metabolism, and oxidative stress.

## 2 Materials and methods

### 2.1 Materials and chemicals

Kaempferol, luteolin and quercetin were purchased from meilunbio (Dalian Meilun Biotechnology Co., Ltd.); Albumin (ALB), SOD and MDA kits were purchased from Nanjing Jiancheng (Nanjing Jiancheng Bioengineering Institute); Tumor necrosis factor-α (TNF-α) and interleukin-6 (Il-6) kits were purchased from Absin (Shanghai Absin Bioscience Inc.); CCl_4_ was purchased from Macklin (Shanghai Macklin Biochemical Technology Co., Ltd.); L-phenylalanine-d5 (Phe-d5, SDHA-013) was purchased from CIL (Andover, United States) and used as an analytical internal standard (IS); N-Ethylmaleimide (NEM, ≥ 99.0%, BCBZ7317), Tris (2-carboxyethyl) phosphine hydrochloride (TECP, ≥ 98.0%, SLBZ2552) was purchased from Sigma-Aldrich (St. Louis, MO, United States); Anti-COX2 Antibody was purchased from BIOSS (Beijing BIOSYNTHESIS Biotechnology CO., LTD.); Anti-α-SMA Antibody from CST (Cell Signaling Technology, Inc.); Anti-TGF-1β Antibody from SAB (Signalway Antibody LLC.); Goat anti-Mouse IgG (H + L) Secondary Antibody, Goat anti-Rabbit IgG (H + L) Secondary Antibody were purchased from CWBIO (Beijing Kangwei Century Biotechnology Co., Ltd.).

### 2.2 Animals

Male SD rats (200–220 g) were purchased from the Guangzhou Ruige Biological Technology Co., Ltd. (the medical experimental animal number was SCXK (YUE) 2021-0059; the animal certification number was NO. 44827200000404). The rats were reared in a well-ventilated environment, with a temperature of about 20–25°C, a 12-hour light day and night cycle, free feed and drinking water, and were reared adaptively for 1 week. The experimental protocols were approved by the Animal Care and Use Committee of Guangzhou Medical University (Ethical approval: 2018-051).

### 2.3 Screening of active components and targets of LFP

The chemical components and related targets of LFP were obtained by searching the pharmacological database and literature on traditional Chinese medicine systems (TCMSP, Traditional Chinese Medicine Systems Pharmacology Database and Analysis Platform, https://old.tcmsp-e.com/tcmsp.php). The potential active compounds were screened based on their oral bioavailability and drug-likeness. The threshold was set as oral availability (OB) ≥ 30% and drug-like degree (DL) ≥ 0.18 because ingredients that meet these two criteria are considered potential active ingredients. The relevant target proteins of each active ingredient were screened in the TCMSP database, the gene name corresponding to the target was obtained using the Uniport (http://www.uniprot.org/), and the organism was selected as “human.”

### 2.4 Collection of potential targets against liver fibrosis

“Liver fibrosis” was used as the key word, and the protein targets and corresponding gene names of liver fibrosis were obtained in the GeneCards (https://www.genecards.org/). “Gifts≥48” was selected as the target for the treatment of liver fibrosis. After obtaining the target of the herb-pair on the active ingredient and the target of the disease, a Venn diagram was drawn by intersecting herb-pair active ingredient targets and disease targets based on “gene name.”

### 2.5 Analysis of the main indicators of anti-hepatic fibrosis

The common target information was imported into the STRING database (https://cn.string-db.org/) to construct a protein-protein interaction (PPI) network. High confidence (0.9) was used as the screening condition to filter out the isolated targets, limit the species to humans, and map the PPI network. Cytoscape3.8.0 built-in plug-in MCODE was used to perform module analysis on the PPI network, obtain potential protein function modules, and analyze and describe their functions.

### 2.6 Gene function annotation and kyoto encyclopedia of genes and genomes pathway analyses

To further determine the biological processes and signaling pathways of the target protein genes, the targets of LFP in the treatment of liver fibrosis were entered into Metascape, the species was limited to humans, and *p* < 0.01 was considered statistically significant. Gene Ontology (GO) and Kyoto KEGG pathway enrichment analyses were performed.

### 2.7 Determination of the active ingredient and ratio in LFP by high-performance liquid chromatography

LFP was first weighed in a conical flask, 100 ml of pure water was added, and incubated for approximately 30 min. The mixture was heated in a water bath at 99°C for 2 h, filtered, 100 ml of pure water was added again, and the mixture was heated in a water bath at 99°C for 1 h and filtered. The combined filtrates were concentrated to approximately 10 ml by evaporation. Methanol was added to the extract (ratio 1:10), the mixture was centrifuged at 15,000 rpm for 15 min, and the supernatant was collected. The chromatographic column used was Agilent ZORBAX SB-C18 (5 μm; Agilent Technologies, Santa Clara, CA, United States) using isocratic elution with a mobile phase consisting of 60% methanol and 0.1% formic acid. The flow rate was 1 ml/min, the column temperature was 40°C, and the detection wavelength was 360 nm.

### 2.8 Therapeutic effect of quercetin-luteolin-kaempferol on CCl_4_-Induced liver fibrosis

**FIGURE 1:**
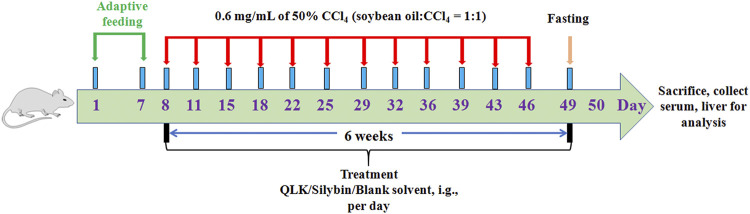
Diagrammatic sketch of the experimental design of the effect of QLK on rat liver fibrosis.

To evaluate the therapeutic effects of the main active ingredients identified extracted from LFP (quercetin, luteolin, and kaempferol), 36 rats were randomly divided into six groups: 1) a control group was administered blank solvent by gavage every day; 2) a CCl_4_-induced rat liver fibrosis group was administered blank solvent by gavage daily; 3) a positive control group was administered 50 mg/kg silybin by gavage daily; 4) a high-dose group (QLKH) was administered 102 mg/kg quercetin, 57 mg/kg luteolin, and 36 mg/kg kaempferol daily by the intragastrical route [i.g.]; 5) a moderate-dose group (QLKM) was administered 51 mg/kg quercetin, 28.5 mg/kg luteolin, and 18 mg/kg kaempferol daily by i.g.; 6) and a low-dose group (QLKL) was administered 25.5 mg/kg quercetin, 14.25 mg/kg luteolin, and 9 mg/kg kaempferol daily by i.g. For groups ii-vi, 0.6 mg/ml of 50% CCl_4_ (soybean oil: CCl_4_ = 1:1) were subcutaneously injected into the rat dorsum twice a week for 6 weeks; and for group i, 0.6 mg/ml of soybean oil were subcutaneously injected into the rat dorsum twice a week for 6 weeks (Figure 1).

#### 2.8.1 Sample collection and liver index calculationauto

Forty-eight hours after the last administration of CCl_4_, the animals were fasted for 24 h, and their body weights were measured and recorded. The animals were then anesthetized with pentobarbital sodium (60 mg/kg) and euthanized. Blood samples were collected from the abdominal aorta for biochemical evaluation. Livers were immediately removed and weighed to calculate the relative liver index (liver index% = liver weight/body weight × 100%). Most of the liver was snap-frozen in liquid nitrogen, and the remaining tissue was fixed with 4% paraformaldehyde and processed.

#### 2.8.2 Histopathological analysis

Fixed livers were embedded in paraffin, and sectioned. Sections were stained with hematoxylin and eosin (H&E), and masson. Histological changes were evaluated by randomly selecting histology using an upright microscope (Nikon, Japan).

#### 2.8.3 Biochemical analysis

The levels of alanine transaminase (ALT), aspartate transaminase (AST), gamma-glutamyl transferase (γ-GGT), total bilirubin (TBIL) were measured using a Chemray 240 Automatic Biochemical Analyzer (Rayto Life and Analytical Sciences Co. Ltd., Shenzhen, China). Albumin (ALB), superoxide dismutase (SOD), and malondialdehyde (MDA) levels were evaluated using commercial kits, according to the manufacturer’s instructions. Tumor necrosis factor-α (TNF-α) and interleukin -6 (IL-6) was detected using commercialized ELISA kits according to the manufacturer’s instructions.

#### 2.8.4 Reverse transcription-polymerase chain reaction

Total RNA was extracted from the liver tissue using RNA extraction solution (Servicebio, Wuhan, China) according to the manufacturer’s instructions. The PCR primer sequences are listed in Table 1. Total RNA (10 µl) was reverse transcribed into cDNA. The PCR reactions (20 µl) included the following reagents: 2 µl cDNA, 1.5 µl of 2.5 µM primers, 7.5 µl of 2 × qPCR Mix, and 4 µl Water Nuclease-Free. The RT-PCR reaction steps were as follows: pre-denaturation at 95°C for 30 s, denaturation at 95°C for 15 s, and annealing at 60°C for 30 s, cycling 40 times. The melting curve was 65–95°C, and the fluorescence signal was recorded once for each increase of 0.5°C. The expression of the mRNAs of α-smooth muscle actin (α-SMA), prostaglandin G/H synthase 2 (COX2), formyl-peptide receptor-2 (FPR2), prostaglandin G/H synthase 1(PTGS1), nuclear receptor coactivator 2 (NCOA2), IL-1β, tumor necrosis factor-α (TNF-α), chemokine [CXC motif] ligand 4 (CXCL14), and transforming growth factor-β1 (TGF-β1), relative to the control gene GAPDH mRNAs, was detected using the 2^−ΔΔCT^ method.

**TABLE 1:** Primers for real-time reverse transcription-polymerase chain reaction (RT-PCR).

Gene	Primer sequences (5′-3′)	Fragment length (bp)
α-SMA	AAC​TGG​TAT​TGT​GCT​GGA​CTC​TG	172
	CTC​AGC​AGT​AGT​CAC​GAA​GGA​ATA	
PTGS2	CGT​TTG​AAG​AAC​TTA​CAG​GAG​AGA​A	94
	AGC​AGG​GCG​GGA​TAC​AGT​T	
IL-1β	TGT​GAC​TCG​TGG​GAT​GAT​GAC	160
	CCA​CTT​GTT​GGC​TTA​TGT​TCT​GTC	
TNF-α	CCA​CCA​CGC​TCT​TCT​GTC​TAC​TG	151
	TGG​GCT​ACG​GGC​TTG​TCA​CT	
PTGS1	CAA​CCT​ACA​ACA​CAG​CAC​ATG​ACT​A	136
	TCT​GGT​AAC​TGC​TTC​TTC​CCT​TT	
FPR2	GTC​CCT​TAC​GAG​TCC​TTA​CAG​CA	198
	ACA​TAG​AGC​ATT​GGA​TTG​AGG​C	
CXCL14	CCA​CAC​TGC​GAG​GAG​AAG​ATG	147
	GTA​GAC​CCT​GCG​TTT​CTC​GTT	
NCOA2	AAC​CAG​CCA​AAC​CAA​CTG​AGA​C	270
	GGT​CAT​ATT​CAA​CCC​TTG​TCC​TCT	
TGF-β1	GCT​GAA​CCA​AGG​AGA​CGG​AAT​A	194
	GCA​GGT​GTT​GAG​CCC​TTT​CC	
GAPDH	CTG​GAG​AAA​CCT​GCC​AAG​TAT​G	138
	GGT​GGA​AGA​ATG​GGA​GTT​GCT	

#### 2.8.5 Western blotting

Proteins were extracted and quantified using the BCA method. The protein samples were subjected to electrophoresis, transferred to a membrane, blocked, and incubated with the following primary antibodies: anti-COX2 (1:1000), anti-TGF-1β (1:1000), anti-α-SMA (1:1000). The membranes were then washed thrice with 1 × tris-buffered saline, 0.1% Tween (TBST), incubated with horseradish peroxidase-labeled secondary antibody (1:3000) for 1 h, and washed thrice with TBST. After the ECL chemiluminescence reagent was added, membranes were scanned (Bio-Rad Laboratories, Hercules, CA, United States), and the images were analyzed using ImageJ software.

#### 2.8.6 Detection of glutathione and hemocyanin in serum by high-performance liquid chromatography-mass spectrometry

Serum (100 µl) was added to 20 µl TECP (100 mM) or ddH_2_O solution at the appropriate concentration, and the reduction reaction was performed at room temperature. Next, 100 µl of NEM (50 mM) solution at the pertinent concentration was added to the first step of the reaction mixture, followed by the addition of 500 µl of cold methanol (50 ng/ml of Phe-d5), vortexing for 30 s, and incubation at −20°C for 60 min. The precipitated proteins were centrifuged (5804R, Eppendorf, Hamburg, Germany) at 21,380 g for 15 min at 4°C, and the supernatant (600 µl) was collected and dried using a stream of dry N_2_ at 30°C. The residue was re-dissolved in 10 vol% aqueous methanol (100 µl), and the solution was vortexed for 1 min and centrifuged at 21,380 g for 15 min at 4°C. An 80 µl aliquot of the supernatant was collected for analysis.

Serum extracts were analyzed on a Sciex 4000 Triple Quadrupole HPLC-MS/MS system (AB Sciex, Toronto, ON, Canada) using an ACQUITY UPLC BEH C18 analytical column (1.7 μm, 2.1 × 50 mm; Waters Co., Milford, MA, United States). The mobile phase comprising 0.1% formic acid in water (solvent A) and 0.1% formic acid in methanol (solvent B) was supplied according to the following program: 0–1 min (5%–95% B); 1–2 min (95%–80% B); 2–3 min (80% B); 3–5 min (90%–80% B); 5–8 min (80%–5% B); and 8–10 min (5% B). The injection volume and flow rate were 10 μl and 0.3 ml/min, respectively. The multiple reaction-monitoring parameters are listed in Table 2. All HPLC-MS/MS data were obtained using the Analyst Software (v1.6.2). The results of the methodological validation are shown in the Supplementary Material. The concentrations of GSH and Hcy in serum were evaluated based on the regression equation with a weighting factor (*1/x*), and GSSG = (Total GSH − Free GSH) × 307.33 × 2/612.63.

**TABLE 2:** Mass spectrometric characteristics of GSH and Hcy.

Compound	Q1 Mass (Da)	Q3 Mass (Da)	DP (Volts)	EP (Volts)	CE (Volts)	CXP (Volts)
Hcy-NEM	261.3	56.12	37.13	6.5	25.21	2.36
GSH-NEM	433.1	304.13	51.85	4.98	21.77	4.47
(D5-Phe) IS	171.1	125.22	16.51	10.47	19.14	3.00

### 2.9 Statistical analysis

All experimental data are presented as mean ± SD. Statistical analysis was performed using GraphPad Prism 9.0.0 statistical software (GraphPad Software Inc., San Diego, CA, United States). The Student’s *t*-test was used to compare the means between two datasets, and one-way analysis of variance (ANOVA) was used to compare the means between three or more data points. *p* < 0.05 was considered statistically significant.

## 3 Results

### 3.1 Network pharmacology prediction

#### 3.1.1 Active components and targets of LFP

The chemical components of LFP were screened using the TCMSP database and the potential active compounds were screened according to oral availability (OB) and drug-like degree (DL). Under OB ≥ 30% and DL ≥ 0.18 as the screening conditions, 23 active ingredients of *Lonicerae Japonicae Flos*, corresponding to 449 targets, and 23 active ingredients of *Forsythiae Fructus*, corresponding to 510 targets were identified. After deleting the duplicates, a total of 31 active ingredients were obtained, and the combination had a total of four ingredients (Table 3). After correction using Uniport, 227 targets were identified.

**TABLE 3:** The active components of LFP.

MOL ID	Molecule name	MW	OB%	DL
MOL001494	Mandenol	308.56	42	0.19
MOL001495	Ethyl linolenate	306.54	46.1	0.2
MOL002914	Eriodyctiol (flavanone)	288.27	41.35	0.24
MOL003006	(-)-(3R,8S,9R,9aS,10aS)-9-ethenyl-8-(beta-D-glucopyranosyloxy)-2,3,9,9a,10,10a-hexahydro-5-oxo-5H,8H-pyrano[4,3-d]oxazolo[3,2-a]pyridine-3-carboxylic acid_qt	281.29	87.47	0.23
MOL003014	Secologanic dibutylacetal_qt	384.57	53.65	0.29
MOL002773	β-carotene	536.96	37.18	0.58
MOL003036	ZINC03978781	412.77	43.83	0.76
MOL003044	Chryseriol	300.28	35.85	0.27
MOL003095	5-hydroxy-7-methoxy-2-(3,4,5-trimethoxyphenyl)chromone	358.37	51.96	0.41
MOL003111	Centauroside_qt	434.48	55.79	0.5
MOL003117	Ioniceracetalides B_qt	314.37	61.19	0.19
MOL003128	Dinethylsecologanoside	434.44	48.46	0.48
MOL000358	β-sitosterol	414.79	36.91	0.75
MOL000422	Kaempferol	286.25	41.88	0.24
MOL000449	Stigmasterol	412.77	43.83	0.76
MOL000006	Luteolin	286.25	36.16	0.25
MOL000098	Quercetin	302.25	46.43	0.28
MOL000173	Wogonin	284.28	30.68	0.23
MOL003283	(2R,3R,4S)-4-(4-hydroxy-3-methoxy-phenyl)-7-methoxy-2,3-dimethylol-tetralin-6-ol	360.44	66.51	0.39
MOL003290	(3R,4R)-3,4-bis[(3,4-dimethoxyphenyl)methyl]oxolan-2-one	386.48	52.3	0.48
MOL003295	(+)-pinoresinol monomethyl ether	372.45	53.08	0.57
MOL003306	ACon1_001697	372.45	85.12	0.57
MOL003308	(+)-pinoresinol monomethyl ether-4-D-beta-glucoside_qt	372.45	61.2	0.57
MOL003315	3beta-Acetyl-20,25-epoxydammarane-24alpha-ol	502.86	33.07	0.79
MOL000211	Mairin	456.78	55.38	0.78
MOL003322	FORSYTHINOL	372.45	81.25	0.57
MOL003330	(-)-Phillygenin	372.45	95.04	0.57
MOL003347	Hyperforin	536.87	44.03	0.6
MOL003370	Onjixanthone I	302.3	79.16	0.3
MOL000522	Arctiin	534.61	34.45	0.84
MOL000791	Bicuculline	367.38	69.67	0.88

#### 3.1.2 Potential anti-hepatic fibrosis targets

**FIGURE 2:**
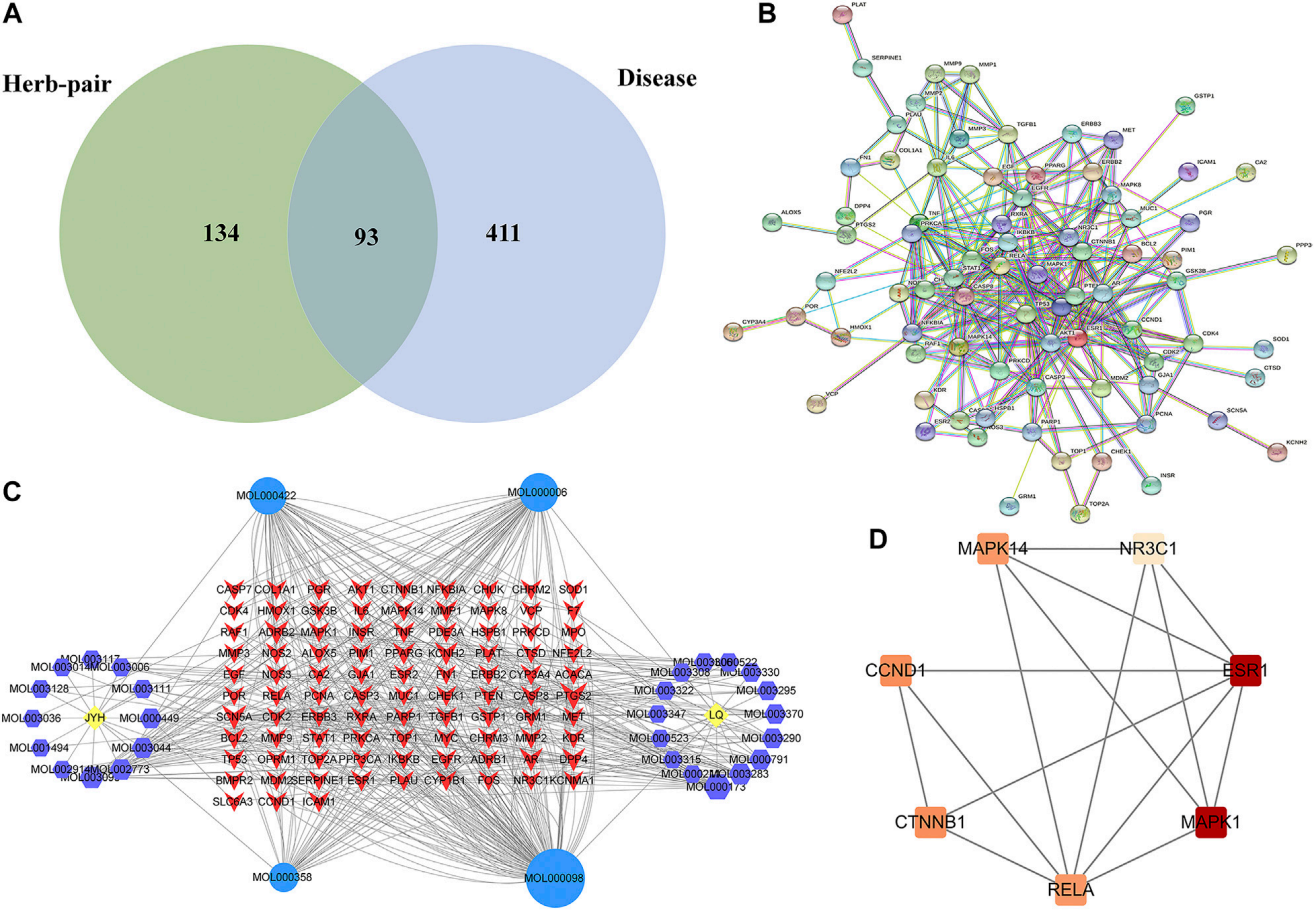
Network pharmacology analysis. **(A)** Venn diagram of LFP and liver fibrosis targets; **(B)** Composite target network of *Lonicerae Japonicae Flos-Forsythiae Fructus* in the treatment of liver fibrosis; **(C)** PPI network; **(D)** Key subnetwork of PPI. JYH, *Lonicerae Japonicae Flos*; LQ, *Forsythiae Fructus*.

In the GeneCards database, using “liver fibrosis” as a keyword, 504 liver fibrosis disease targets with Gifts ≥48 were screened, and the target of the active ingredient of the herb-pair was intersected with the target of the disease. Finally, 93 targets related to honeysuckle-forsythia in the treatment of liver fibrosis were identified (Figure 2A).

**FIGURE 3:**
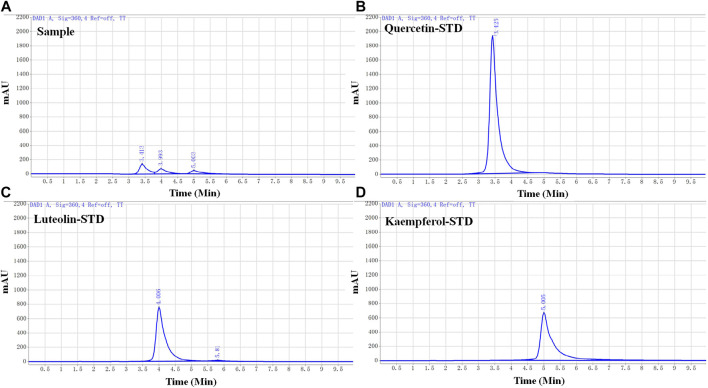
High-performance liquid chromatography (HPLC) chromatograms of honeysuckle-forsythia water extract and each active ingredient. **(A)** HPLC chromatogram of honeysuckle-forsythia water extract. 1: Quercetin, 2: Luteolin, 3: Kaempferol. **(B)** Quercetin. **(C)** Luteolin. **(D)** Kaempferol.

#### 3.1.3 Main indicators of anti-hepatic fibrosis

The medicinal material-ingredient-target network contained 126 nodes and 450 edges. As shown in Figure 2B, two diamond-shaped nodes represent medicinal materials, 30 hexagonal nodes represent independent active ingredients of the two medicinal herbs, four circular nodes represent active ingredients shared by the two medicinal herbs, and 93 V-shaped nodes represent target genes. The size of the node is set according to the degree of connection. The higher the degree value, the larger the node, indicating that the more nodes an ingredient connects to in the network, the more important its role. The results show one compound acting on multiple targets in LFP and different compounds acting on the same target, which reflects the multi-component and multi-target action characteristics of TCM. According to the network topology parameters, the main active ingredients were quercetin, kaempferol, and luteolin (Table 4), and the core targets were PTGS2, SCN5A, ADRB2, AR, and DPP4 (Table 5).

**TABLE 4:** The node characteristic parameters of main active ingredients in the compound-target.

Mol ID	Component name	Degree	Betweenness	Closeness
MOL000098	Quercetin	119	0.4661	0.5605
MOL000006	Luteolin	58	0.1274	0.4417
MOL000422	Kaempferol	54	0.1121	0.4325

**TABLE 5:** The node characteristic parameters of the main targets in the compound-target network.

Target	Target name	Degree	Betweenness	Closeness
PTGS2	Prostaglandin G/H synthase 2	25	0.1129	0.5187
SCN5A	Sodium channel protein type 5 subunit alpha	17	0.0447	0.4579
ADRB2	Beta-2 adrenergic receptor	15	0.031	0.4417
AR	Androgen receptor	13	0.0373	0.4545
DPP4	Dipeptidyl peptidase IV	13	0.0265	0.4417

**TABLE 6:** The calibration curves and linearity range of the three main compounds.

Compounds	Regression equation	*R* ^2^	Linearity range(μg/mL)
Quercetin	*Y* = 185.12*X*-32.474	0.9998	0.2–40
Luteolin	*Y* = 245.16*X*-70.408	0.9992	0.1–40
Kaempferol	*Y* = 208.44*X*-23.424	0.9999	0.2–40

A PPI network containing 93 targets and 299 interaction lines was obtained using the STRING database to analyze the interactions of 93 drug-disease intersection targets and by setting high confidence (0.9) and hiding isolated targets (Figure 2C). The PPI network analysis revealed that TP53, MAPK1, ESR1, AKT1, and CTNNB1 ranked in the top 5% of predicted proteins. Using the MCODE plug-in in Cytoscape 3.8.0, module analysis of the PPI network was performed, and a module with a K-core value greater than four was obtained. (Figure 2D).

#### 3.1.4 GO analysis and KEGG analysis

GO and KEGG analyses were performed using the Metascape platform. A total of 5676 GO items were obtained, including 395 for cellular composition, 645 for molecular function, and 4636 for biological processes. The enrichment results showed that the targets of the LFP were mainly distributed in the membrane raft, vesicle lumen, and transcription regulator complex (Supplementary Figure S1A), the main molecular functions of which were protein kinase activity, kinase binding, and transcription factor binding (Supplementary Figure S1B). The biological processes involved mainly included pathways related to cancer, the IL-18 signaling pathway, and response to inorganic substances (Supplementary Figure S1C). A total of 240 KEGG signaling pathways were analyzed, the most notable of which were pathways related to cancer, prostate cancer, and the HIF-1 signaling pathway (Supplementary Figure S1D).

### 3.2 Determination of the active ingredient ratio in LFP by HPLC.

Three active ingredients (quercetin, luteolin, and kaempferol) of LFP were selected for further animal studies based on the previous database analysis and a literature search. To determine the ratio of the active ingredients in the drug combination, the standard calibration curves and linearity range of quercetin, luteolin, and kaempferol were investigated (Table 6), and the water extracts of LFP were analyzed by HPLC (Figure 3).

The concentrations of quercetin, luteolin, and kaempferol (QLK) in LFP were 16.84 μg/g, 9.36 μg/g, and 6.09 μg/g, respectively. The ratio of quercetin: luteolin: kaempferol was 34:19:12. Based on this ratio, we designed a dosing regimen for the subsequent animal experiments.

### 3.3 QLK protects against hepatic fibrosis in rats.

To evaluate the therapeutic effects of QLK, 36 rats were randomly divided into six groups: 1) a control group that was administered blank solvent; 2) a CCl_4_-induced rat liver fibrosis model group was administered blank solvent; 3) a positive control group that was administered 50 mg/kg silybin; 4) a high-dose group (QLKH) that was administered high doses of QLK; 5) a moderate-dose group (QLKM) that was administered moderate doses of QLK; 6) and a low-dose group (QLKL) that was administered low doses of QLK. For groups ii-vi, 0.6 mg/ml of 50% CCl_4_ was subcutaneously injected into the rat dorsum twice a week for 6 weeks; and for group i, 0.6 mg/ml of soybean oil was subcutaneously injected into the rat dorsum twice a week for 6 weeks.

The rats were then sacrificed, blood samples were collected from the abdominal aorta for biochemical evaluation, and the livers were immediately removed for histopathological analysis.

**FIGURE 4:**
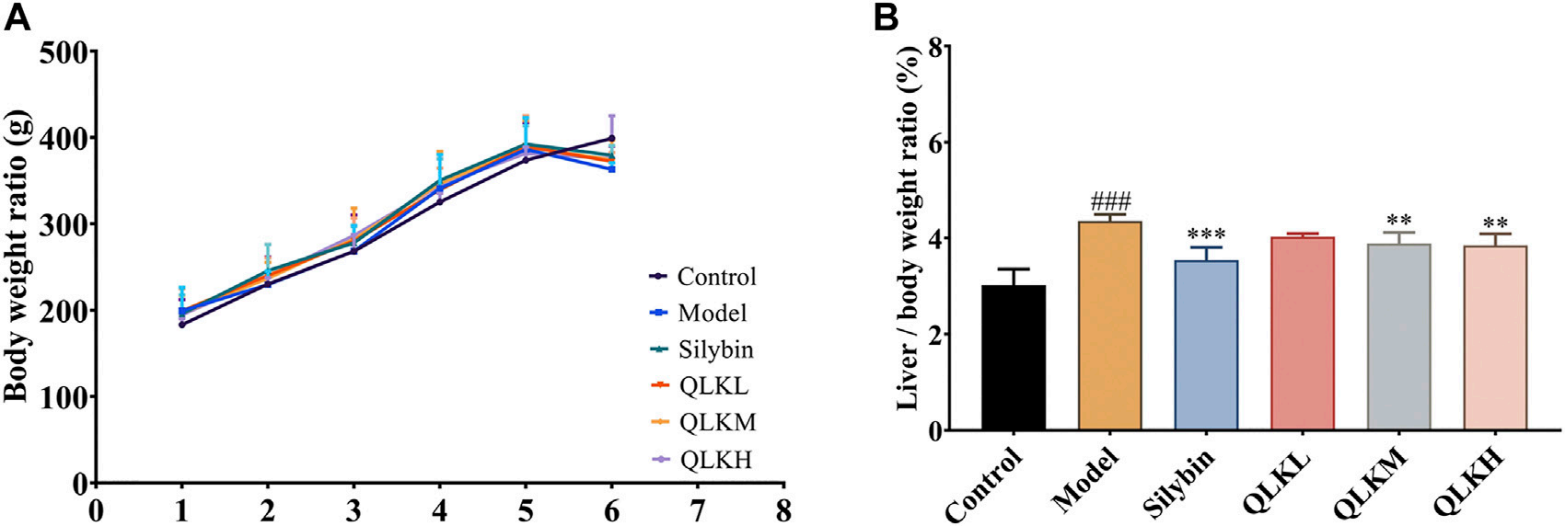
Effect of QLK treatment on the body weight and liver index in the CCl_4_ rats (*n* = 6). **(A)** Body weight for 6 weeks **(B)** Liver index. Values are presented as mean ± SD for three biological replicates per group. **p < 0.01 vs. model, ***p < 0.005 vs. model, ^###^ indicates *p* < 0.001 vs. control. QLK, quercetin-luteolin-kaempferol.

The liver index of the model group was higher than that of the control group. The liver indices of the positive control, QLKM, and QLKH groups were all significantly decreased (*p* < 0.05) compared to that of the model group (Figure 4).

**FIGURE 5:**
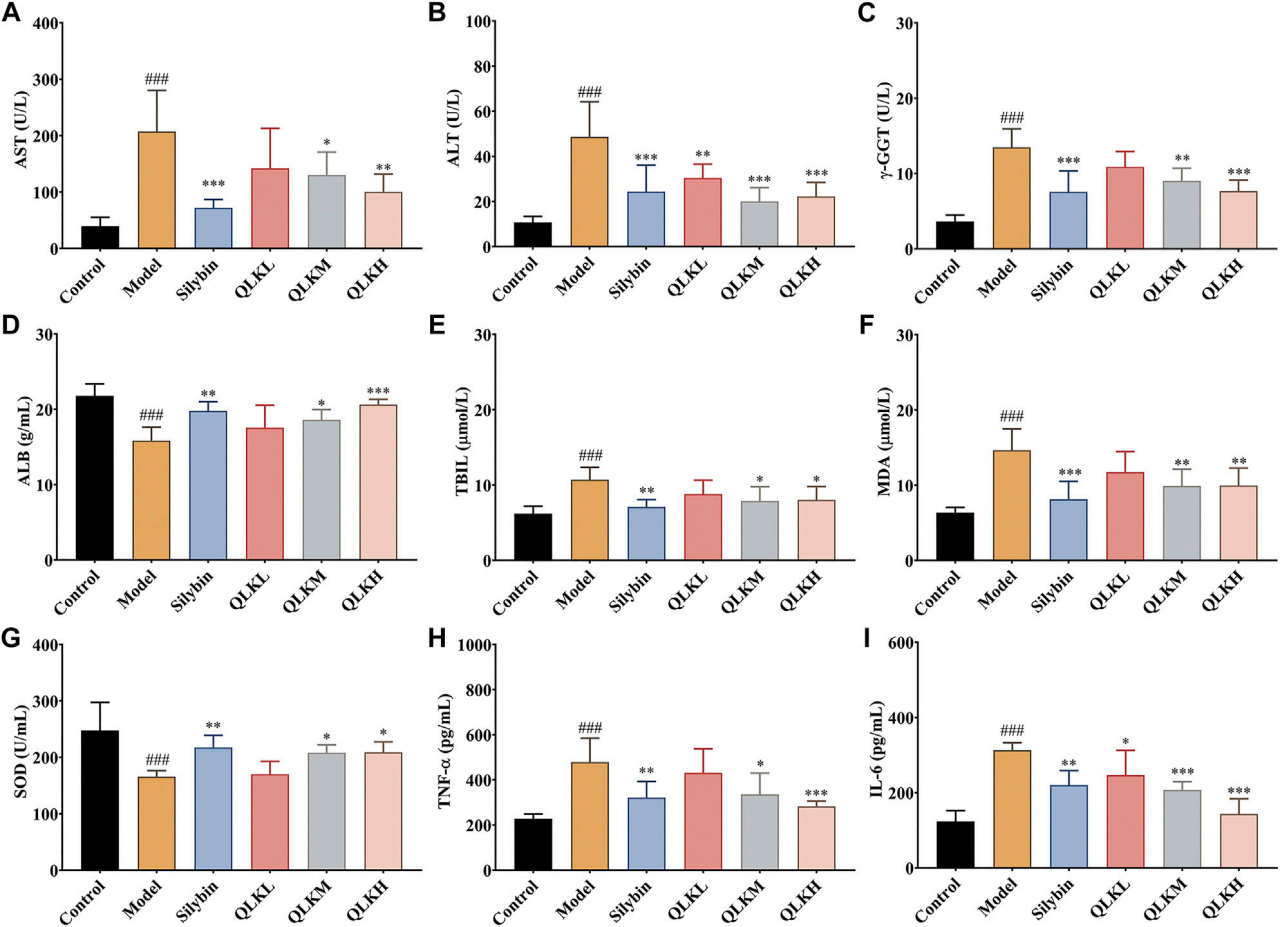
Effect of QLK treatment on the levels of serum biochemical indices in CCL_4_ rats (*n* = 6). **(A)** Serum AST; **(B)** Serum ALT; **(C)** Serum γ-GGT; **(D)** Serum ALB; **(E)** Serum TBIL; **(F)** Serum MDA; **(G)** Serum SOD; **(H)** Serum TNF-α; **(I)** Serum IL-6; Values are presented as mean ± SD for three biological replicates per group. *p < 0.05, **p < 0.01, ***p < 0.005 vs. model, ^###^ *p* < 0.005 vs. control. QLK, quercetin-luteolin-kaempferol.

In addition, serum ALB levels were significantly reduced in the model group (*p* < 0.05), whereas serum ALT, AST, TBIL, and γ-GGT levels, which were used to evaluate hepatocellular necrosis and cholestasis associated with hepatic fibrosis, were drastically increased compared to the control group. Interestingly, QLK treatment reduced serum ALT, AST, TBIL, and γ-GGT levels in a dose-dependent manner, alleviated CCl_4_-induced hepatic oxidative stress, increased serum SOD levels, and decreased MDA levels. QLK treatment also alleviated CCl4-induced hepatic inflammation and decreased TNF-α and IL-6 levels (Figure 5).

#### 3.3.1 QLK alleviates liver fibrosis

The effectiveness of QLK in alleviating CCl_4_-induced rat liver fibrosis was evaluated using H&E and Masson staining. The histopathologic analysis showed that the livers of the control group had smooth and soft surfaces, whereas those of CCl_4_-induced fibrosis groups exhibited a fibrous network and adhesions covering the surface.

**FIGURE 6:**
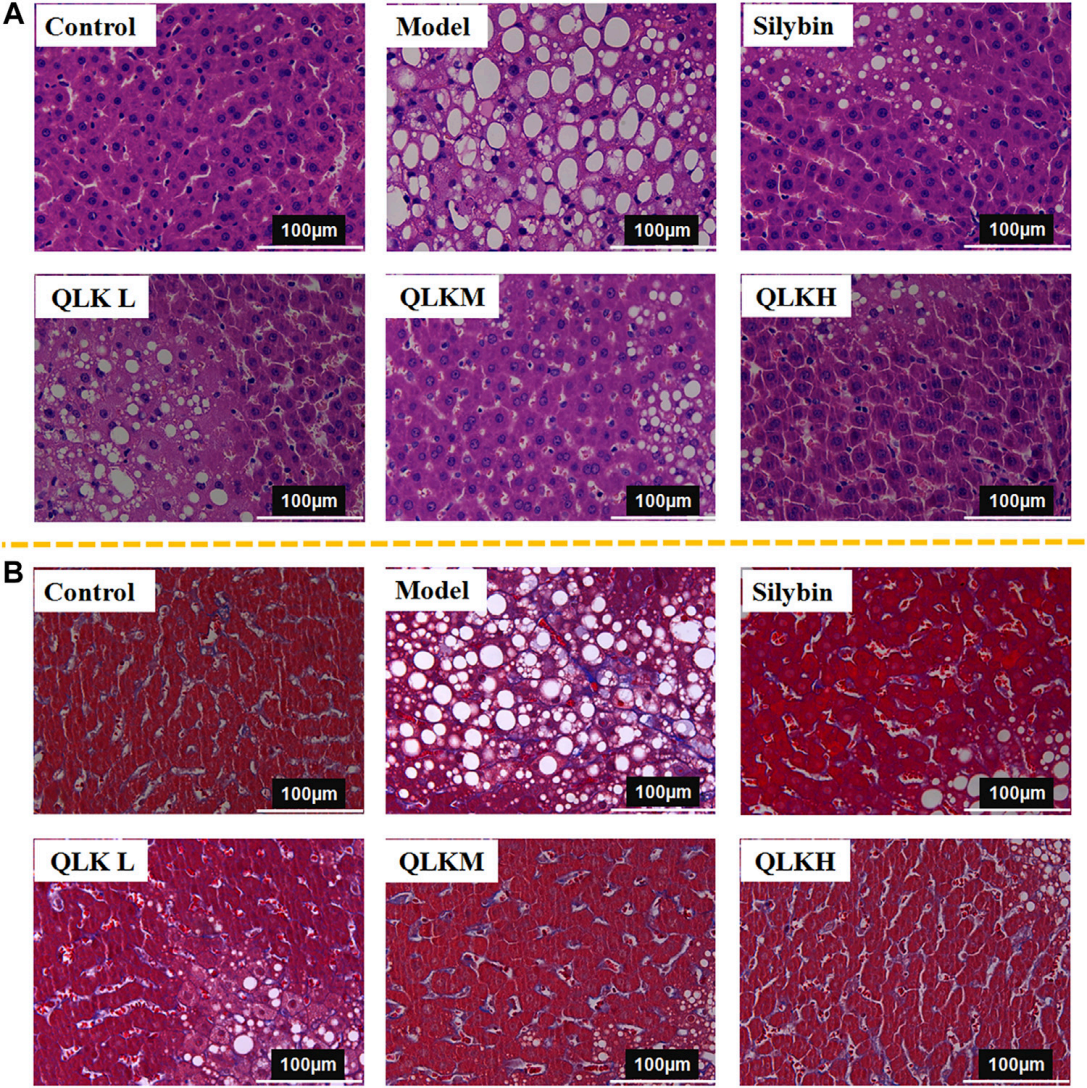
Histological analysis. **(A)** Hematoxylin and eosin (H&E)-stained sections of the liver. Original magnification × 40. **(B)** Masson-stained sections of the liver. Original magnification × 40.

H&E staining (Figure 6A) showed that the hepatic lobule structure of the rats in the control group was normal, the structure of the liver cells was complete, the size was uniform, and the nucleus was clearly visible without inflammation, necrosis, and fibrosis. In the model group, the hepatocytes of the rats were different in size, the cytoplasm was loose, vacuoles of different sizes were present in the cells, and the liver tissue was infiltrated by inflammatory cells, accompanied by balloon-like deformation of the cells.

Masson staining (Figure 6B) revealed that the liver tissue sections of the control group showed a complete cellular structure and no obvious blue collagen fiber deposition, whereas those of the model group showed abnormally structured hepatic lobules, showing degeneration and necrosis of liver cells with massive blue collagen fibril deposition in the liver, indicating severe fibrosis. The Area of collage fibers in liver according to masson stain was shown in Supplementary Figure S2.

Notably, QLK treatment significantly attenuated the degree of hepatic putrescence, fibrosis, and inflammatory cell infiltration, indicating a dose-effect relationship. These results revealed that QLK protected against CCl_4_-induced liver fibrosis in rats.

#### 3.3.2 QLK inhibits fibroblast differentiation and inflammatory reactions, promotes energy balance, and regulates lipid metabolism

**FIGURE 7:**
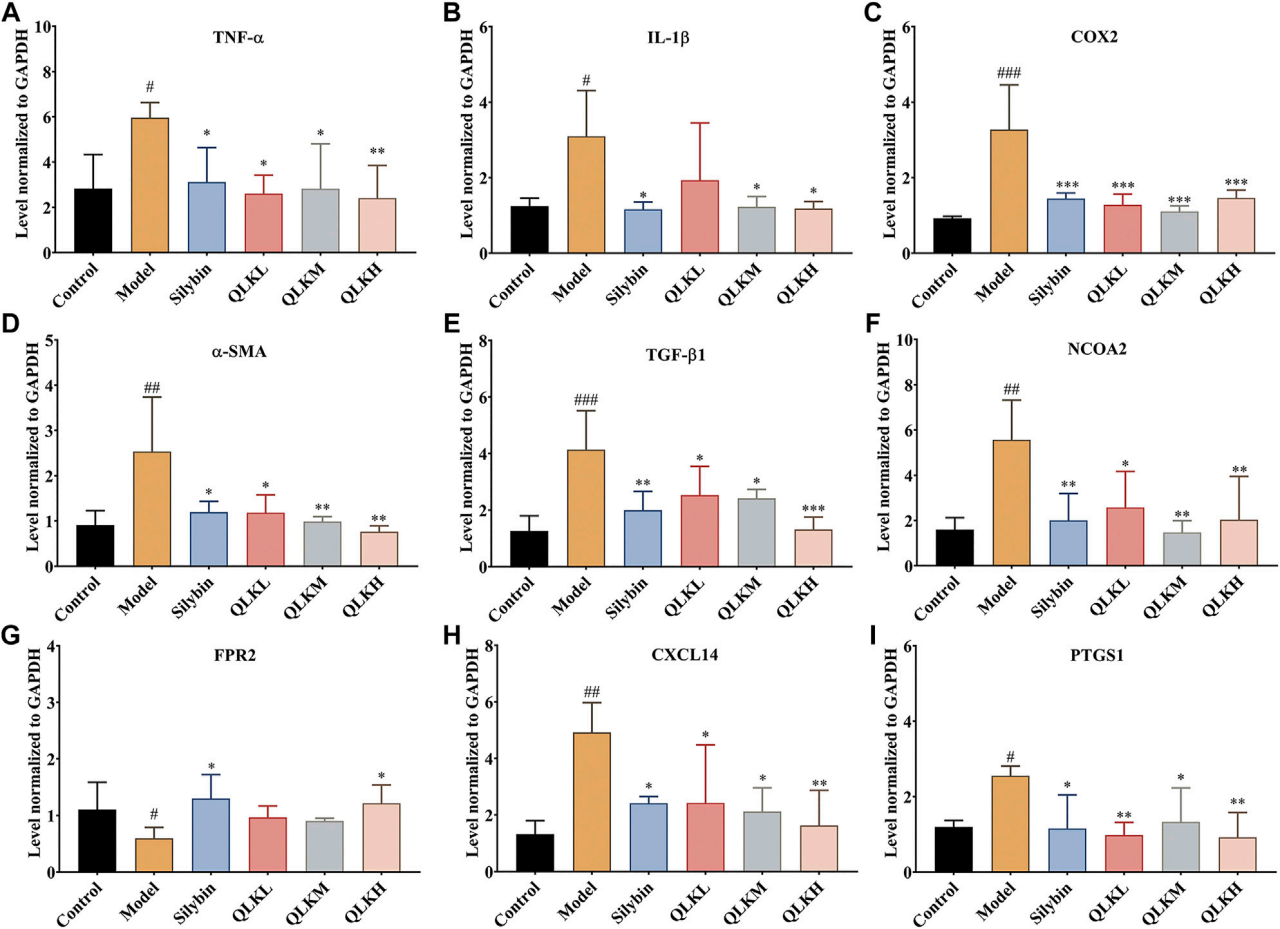
RT-PCR analysis for TNF-α, IL-1β, COX2, α-SMA, TGF-β1, NCOA2, FPR2, CXCL14 mRNA levels in the liver of model rats (*n* = 6). **(A)** TNF-α; **(B)** IL-1β; **(C)** COX2; **(D)** α-SMA; **(E)** TGF-β1; **(F)** NCOA2; **(G)** FPR2; **(H)** CXCL14; **(I)** PTGS1; Values are presented as mean ± SD for three biological replicates per group. *p < 0.05, **p < 0.01, ***p < 0.005 vs. model, ^#^ *p* < 0.05, vs. control, ^##^ *p* < 0.01 vs. control, ^###^ *p* < 0.005 vs. control.

To investigate the underlying mechanism of the protective effect of QLK against liver fibrosis, mRNA levels of α-SMA, COX2, FPR2, PTGS1, NCOA2, IL-1β, TNF-α, CXCL14, and TGF-β1 were measured using RT-PCR. Compared to the control group, α-SMA, COX2, FPR2, PTGS1, NCOA2, IL-1β, TNF-α, CXCL14, and TGF-β1 were upregulated, and FPR2 was downregulated in the model group (Figure 7). This suggests that QLK reduces inflammation, thereby protecting the liver. The expression of FPR2 mRNA was higher than in the CCl_4_-induced model group. These results revealed that QLK protects the liver by inhibiting fibroblast differentiation, inflammatory reactions, promoting energy balance, and regulating lipid metabolism.

#### 3.3.3 QLK reduces collagen formation and inflammatory responses

**FIGURE 8:**
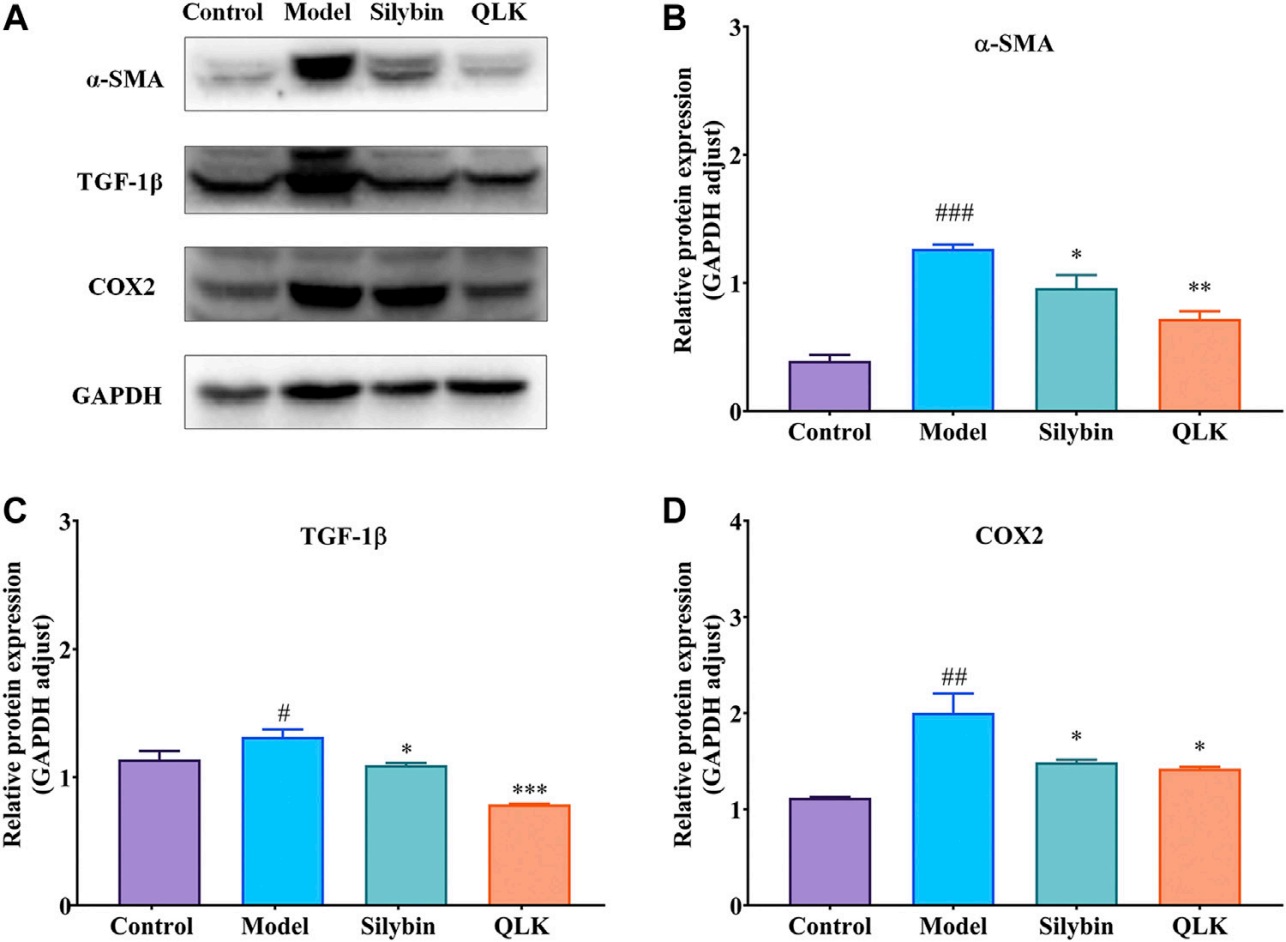
Western blot analysis for α-SMA, TGF-1β, and COX2 protein levels in the liver of model rats (*n* = 3). **(A)** Western blot analysis of α-SMA, TGF-1β, and COX2 protein levels; **(B)** Relative protein of α-SMA was quantitatively expressed by densitometric analysis, GAPDH was served as an internal control; **(C)** Relative protein of TGF-1β; **(D)** Relative protein of COX2. Values are presented as mean ± SD for three biological replicates per group. *p < 0.05, **p < 0.01, ***p < 0.005 vs. model, ^#^ *p* < 0.05, vs. control, ^##^ *p* < 0.01 vs. control, ^###^ *p* < 0.005 vs. control. α-SMA, α-smooth muscle actin; TGF-1β, transforming growth factor 1β; COX2, cyclooxygenase-2.

To further evaluate the therapeutic effect of QLK on the CCl_4_-induced liver fibrosis rat model, the expression of α-SMA, TGF-1β, and COX2 was detected by western blotting. The results showed that compared with the control group, the expression levels of α-SMA, TGF-1β, and COX2 were increased in the model group, whereas QLK administration reduced their expression levels (Figure 8). These results indicated that QLK can protect the liver cells by reducing collagen formation and inhibiting the inflammatory response.

#### 3.3.4 QLK ameliorates serum biomarkers of inflammation and oxidative stress

**FIGURE 9:**
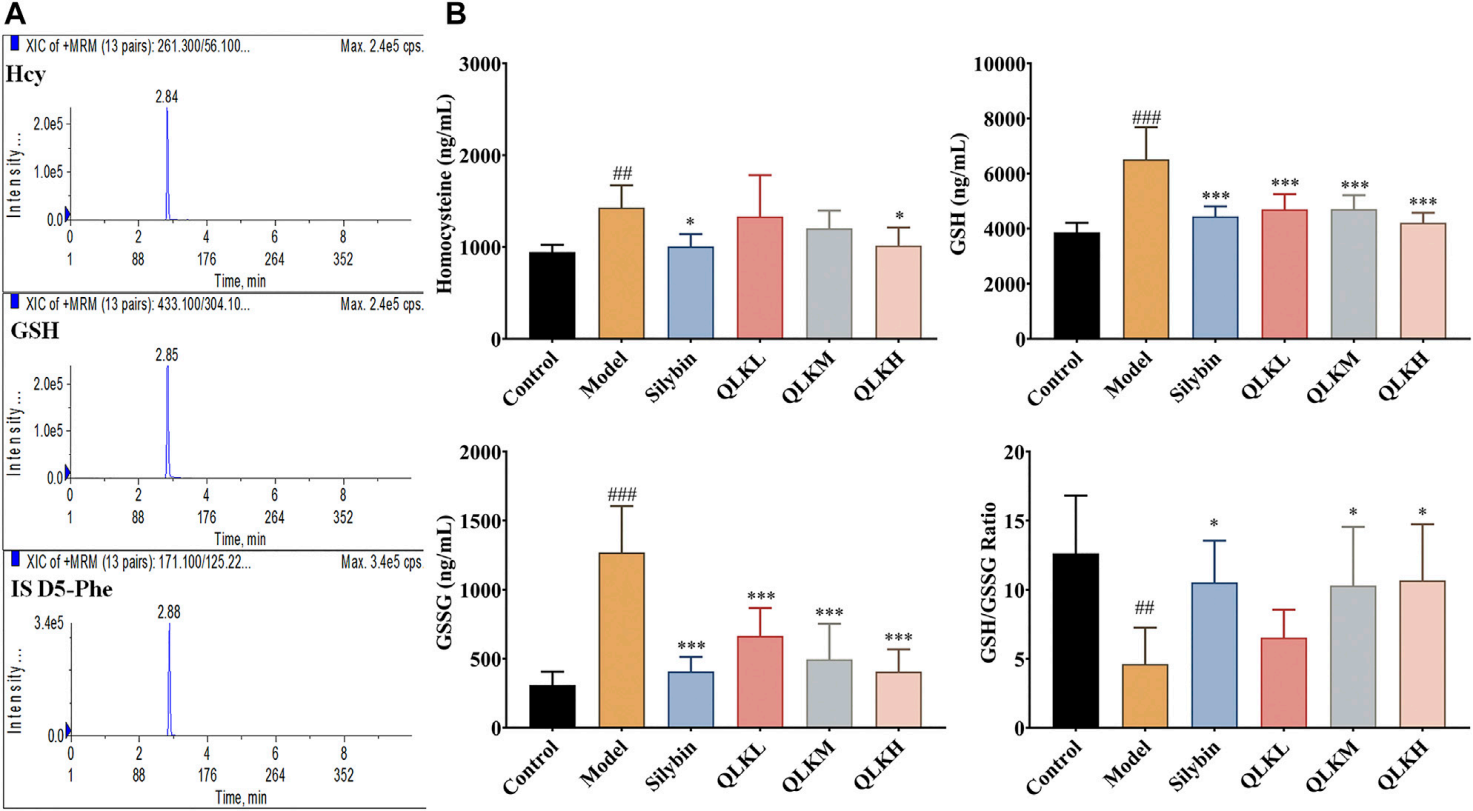
HPLC-MS/MS analysis for serum Hcy, GSH, and GSSG levels in model rats (*n* = 6). **(A)** Typical chromatograms of Hcy, GSH, and internal standard (D5-Phe); **(B)** Serum Hcy, GSH, GSSG levels, and GSH/GSSG ratio in model rats. Values are presented as mean ± SD for three biological replicates per group.*p < 0.05, ***p < 0.005 vs. model; ^##^ *p* < 0.01 vs. control, ^###^ *p* < 0.005 vs. control. Hcy, homocysteine; GSH, reduced glutathione.; GSSG, oxidized glutathione.

Reduced glutathione (GSH) and homocysteine (Hcy) levels reflect the degree of liver injury caused by inflammation and oxidative stress. Therefore, we detected the serum GSH and Hcy levels by LC/MS, and the results showed that the model group had higher GSH, GSSG and Hcy levels than the control group, and lower GSH/GSSG ratio. Notably, QLK treatment reduced GSH, GSSG and Hcy levels, and raised the GSH/GSSG ratio (Figure 9), indicating that QLK can reduce oxidative stress levels and liver damage.

## 4 Discussion

In this study, we combined network pharmacology and animal experiments to construct a “drug component-disease target” network by screening the main active components and targets of *Lonicerae Japonicae Flos* and *Forsythiae Fructus* for the treatment of liver fibrosis. We provided a possible molecular mechanism of the protective effect of LFP and verified its targets and mechanisms using a CCl_4_-induced rat liver fibrosis model. The CCl_4_ has a wide range of hepatotoxicity and is in many ways similar to human chronic diseases. However, due to its high toxicity, it is necessary to pay attention to the concentration and administration time during operation. In this research, the designed dosage and frequency of administration can ensure a better incidence of liver fibrosis, and assure the survival rate of experiment animals.

The main chemical components of *Lonicerae Japonicae Flos* include flavonoids, iridoids, organic acids, and saponins, and the main chemical components of *Forsythiae Fructus* include alkaloids, flavonoids, phenethyl glycosides, triterpenes, and lignans (Guo et al., 2015). LFP extracts have various biological activities, including anti-inflammatory, antioxidative, and antitumor effects (Gao et al., 2014; Bao et al., 2016; Zhou et al., 2017; Lin et al., 2021). Li et al. (2013) have revealed that LFP synergistically reduced inflammatory injury in a rat model of chronic obstructive pulmonary disease. Quercetin, luteolin, and kaempferol identified in the LFP extracts are flavonoids, and flavonoids have been extensively studied in the treatment of liver fibrosis diseases (Li et al., 2015; Wu et al., 2017; Li et al., 2018; Xu et al., 2019; Hu et al., 2020b). In the present study, we showed that QLK reduced the degeneration and necrosis of liver cells caused by CCl_4_ and alleviated the degree of inflammation and liver damage.

Activated hepatic stellate cells (HSCs) play a crucial role in the deposition of fibrotic tissues during liver fibrosis and cirrhosis (Gao et al., 2020). After liver injury, HSCs, the major collagen-synthesizing cells in the liver, are activated and transformed into myofibroblast-like cells, which promote cell proliferation and survival, chemotaxis, and collagen production. HSC activation is driven by a variety of mediators, such as chemokines, reactive oxygen species, and growth factors (She et al., 2020). α-SMA and TGF-β1 are markers of HSC activation (Mederacke et al., 2013). In our study, QLK reduced the protein expression levels of both α-SMA and TGF-β1, indicating that QLK can inhibit HSC activation and reduce collagen production.

We are concerned about that PRMT5 plays an important role in human cancers by promoting the cell proliferation, invasion, and migration, besides, PRMT5 inhibition was efficacious ameliorate leukocyte and platelet counts, hepatosplenomegaly, and fibrosis in the MPLW515L model of myelofibrosis. In addition, bromodomain-containing proteins (CPI-0610) combine with janus kinase inhibitor (ruxolitinib)for myelofibrosis that provide valuable ideas for the treatment of liver disease. We intend to design relevant research on the liver fibrosis model (Pastore et al., 2020; Chen et al., 2021a; Wang et al., 2021).

Hepatic inflammation is an important factor that leads to liver damage. In our study, the results of network pharmacology indicated that the main relevant targets of LFP in the treatment of CCl_4_-induced liver fibrosis include PTGS2. PTGS2, also known as COX2, is an enzyme that is activated by various inflammatory factors, such as cytokines and bacteria. Under inflammatory stimuli, COX2 promotes inflammation and the synthesis of prostaglandins. COX2 has been also reported to play a crucial role in the occurrence and development of liver fibrosis (Yang et al., 2020). In addition, the reduction in the levels of PTGS1, a key enzyme in prostaglandin biosynthesis, also indicates a reduction in inflammation. In this study, we explored the anti-hepatic fibrosis mechanism of QLK by studying other inflammatory biomarkers. The results showed that QLK reduced the expression of pro-inflammatory cytokines, including IL-1β, TNF-α, and CXCL14, and increased the expression of FPR2. IL-1β can reduce the expression of α-SMA in HSCs (Meier et al., 2019), and may prevent the progression of liver injury and fibrosis in various liver pathologies driven by NLRP3 activation (Wree et al., 2018). CXCL14 is a recently discovered member of the CXC chemokine family (Lu et al., 2016), whose overexpression remarkably aggravates CCl_4_-induced liver injury, making it a potential therapeutic target for liver fibrosis (Li et al., 2011; Wang et al., 2020). FPR2 is mainly expressed in human immune cells and is involved in the occurrence and development of various inflammatory diseases. FPR2 is activated by endogenous proteins and lipid ligands, alleviates inflammatory responses, and plays protective roles in the body.

NCOA2, also known as steroid receptor coactivator 2 (SRC-2) (Dasgupta et al., 2015; Cai et al., 2019), plays an important role in lipogenesis, lipid metabolism, and the control of bile release from the liver. NCOA2 downregulation reduces fat accumulation and serum lipid levels in mice (O’Malley, 2020). Furthermore, NCOA2 is associated with fibrosis and is essential for the epithelial-mesenchymal transformation in breast cancer cells (Chopra et al., 2011; Bozickovic et al., 2019; Yang et al., 2021). Oxidative stress can induce the expression of GSH synthase (Lu, 2009). GSH is an important intracellular antioxidant and redox potential regulator and plays an important role in the detoxification and elimination of drugs and the protection of cells against damage by free radicals, peroxidic substances, and toxins (Wu and Batist, 2013). Chemical hepatotoxins decrease the activity of GSH peroxidase, the major antioxidant enzyme present in the liver, and interfere with the oxidation of GSH into GSSG. In addition, serum GSH levels may compensate for the increase in the stage of liver fibrosis (George et al., 2019). In this study, serum GSH and GSSG levels were increased in the liver fibrosis rat model, whereas the GSH/GSSG ratio was decreased. QLK treatment reduced serum GSH and GSSG levels, suggesting that QLK can enhance the activity of glutathione peroxidase, ameliorate oxidative stress, and reduce liver cell damage.

In addition, methionine metabolism plays a pivotal role in liver fibrosis (Li et al., 2020). Hcy is an important intermediate of methionine metabolism. Hcy induces liver fibrosis by stimulating the expression of tissue inhibitor of metalloproteinase-1 (TIMP-1) and type I procollagen (García-Tevijano et al., 2001). TIMP-1 is an important component of the extracellular matrix regulatory system and an inhibitor of matrix metalloproteinases, which are involved in collagen degradation. Moreover, Hcy promotes the overexpression of α-SMA, proliferating cell nuclear antigen (PCNA), and transforming growth of TGF-β1 (Cao et al., 2013). These results indicate that Hcy plays an important role in the process of liver fibrosis. In our study, QLK administration reduced Hcy levels, indicating that QLK can effectively reduce collagen deposition, inhibit HSC proliferation, and alleviate liver damage.

Lipid peroxidation is an important pathological mechanism underlying CCl_4_-induced liver damage (Yu et al., 2020). The liver is the only organ in the human body that synthesizes ALBs; therefore, ALB levels reflect the anabolic and reserve functions of the liver. The severity of liver cirrhosis is a prognostic indicator of liver fibrosis, and a decrease in ALB levels indicates liver function damage. In addition, following liver injury, the cell membranes of the liver cells become damaged, leading to edema and degeneration of liver cells. Additionally, intracellular ALT, AST, and γ-GGT levels are released into the serum after liver injury; therefore, their serum levels reflect the degree of liver injury. Our results showed that QLK reduced the serum levels of ALT, AST, and γ-GGT, indicating that the treatment alleviated the extent of liver damage.These results suggest that the relief of liver cell damage by QLK is associated with free radical scavenging, inhibition of lipid peroxidation, and reduction in the production of lipid peroxides.

In this study, we showed that QLK, a combination of two herbal TCM, alleviates the symptoms of CCl_4_-induced liver fibrosis in rats. However, the mechanism of liver fibrosis alleviation by QLK needs to be further explored and verified in subsequent studies.

## 5 Conclusion

In summary, this study revealed that quercetin, luteolin, and kaempferol are the main active ingredients of LFP that mediate its effects in the treatment of liver fibrosis. QLK can activate FPR2; inhibit α-SMA, COX2, FPR2, PTGS1, NCOA2, IL-1β, TNF-α, CXCL14, and TGF-β1; and reduce collagen formation, fibroblast formation, inflammatory responses, and oxidative stress. Furthermore, LFP plays a hepatoprotective role by inhibiting hepatocyte apoptosis and ballooning degeneration. The results of this study may provide support for future research and clinical application on the role of QLK in human liver fibrosis.

## Data Availability

The original contributions presented in the study are included in the article/Supplementary Material, further inquiries can be directed to the corresponding authors.
