# Supplementary material for: The combination of Lonicerae Japonicae Flos and Forsythiae Fructus herb-pair alleviated inflammation in liver fibrosis
Source: Front Pharmacol. 2022 Aug 19;13:984611. doi: 10.3389/fphar.2022.984611 (PMC9437263; doi:10.3389/fphar.2022.984611)
Supplement: Supplementary file 1 [file DataSheet1.zip › Supplementary material-The original results of Methodological evaluation and Western blot and figure legend--Revised version/Supplementary_Material_Template.docx]

The Combination of *Lonicerae Japonicae Flos* and *Forsythiae* *Fructus* Herb-Pair Alleviated Inflammation in Liver Fibrosis

**Hong-liu Jin^1,2,3, #^, Jing-bei Zhang^4, #^, Xiao-ying Feng^1,2,3^, Sen-lin Feng^1,2,3^, Wen-ting Zhu^1,2,3^, Hong-mei Nan^5,*^, Zhong-Wen Yuan^1,2,3,**^**

^1^ Department of Pharmacy, The Third Affiliated Hospital of Guangzhou Medical University, Guangzhou, China.

^2^ School of Pharmaceutical Sciences, Guangzhou Medical University, Guangzhou, China.

^3^ Guangdong Provincial Key Laboratory of Major Obstetric Diseases, Guangzhou Medical University, Guangzhou, China.

^4^ Collage of Chinese medicine, Changchun University of Chinese Medicine, Jingyue National High-thec Industrial Development,Changchun, Jilin.

^5^ Affiliated Hospital of Changchun university of Chinese medicine, Gongnong Road,Chaoyang district, Changchun, Jilin.

# Equal contributors Hong-liu Jin and Jing-bei Zhang contributed equally to the work.

Corresponding authors:

*** Correspondence:**

*Hong-mei Nan, Affiliated Hospital of Changchun University of Chinese Medicine, 1478#, Gongnong Road, Chaoyang district, Changchun 130117, Jilin, China. nhm19710315@163.com

**Zhong-Wen Yuan, Department of Pharmacy/ Guangdong Provincial Key Laboratory of Major Obstetric Diseases, The Third Affiliated Hospital of Guangzhou Medical University, 63#, Duobao Street, Guangzhou 510150, Guangdong, China. zhwyuan1985@163.com.

Supplementary Material

# Supplementary Data

**1.1 Methodological evaluation**

Supplementary Table 1. Calibration curves in 3 different days in 5% BSA.

| Cpd. |  | Standard curves | R^2^ | Ranger |
| --- | --- | --- | --- | --- |
|  | Day 1 | *y = 0.0017 x + 0.0146* | 0.9994 |  |
| HCY | Day 2 | *y = 0.00177 x - 0.01007* | 0.9996 | 20-4000 ng/ml |
|  | Day 3 | *y = 0.0019 x - 0.0586* | 0.9993 |  |
|  | Day 1 | *y = 0.0002 x + 0.0001* | 0.9995 |  |
| GSH | Day 2 | *y = 0.00021 x + 0.00406* | 0.9994 | 20-4000 ng/ml |
|  | Day 3 | *y = 0.000223 x - 0.000456* | 0.9998 |  |

Supplementary Table 2. Recovery and within-run and between-run precision and accuracy values in 5% BSA (n = 3).

|  |  | Recovery  (%, x±S.D.) | Intra-day | | Inter-day | |
| --- | --- | --- | --- | --- | --- | --- |
|  |  |  | Accuracy (%) | Precision (%) | Accuracy (%) | Precision (%) |
| HCY | 0.4 | 104.17±7.93 | 97.09±5.64 | 5.81 | 101.26±6.29 | 6.21 |
|  | 40 | 103.21±5.40 | 100.14±6.97 | 6.96 | 101.90±6.97 | 6.84 |
|  | 200 | 105.46±2.51 | 95.80±3.70 | 3.86 | 95.40±3.91 | 4.10 |
|  | 1000 | 94.23±6.78 | 101.07±4.04 | 3.99 | 98.08±5.66 | 5.77 |
| GSH | 0.4 | 97.38±4.96 | 107.51±5.76 | 5.36 | 107.78±6.66 | 6.18 |
|  | 40 | 107.57±4.09 | 96.75±5.75 | 5.94 | 95.05±5.32 | 5.60 |
|  | 200 | 100.63±2.99 | 95.71±7.15 | 7.47 | 97.11±6.18 | 6.37 |
|  | 1000 | 99.39±3.84 | 96.15±7.35 | 7.65 | 95.81±5.62 | 5.86 |

Supplementary Table 3. Stability testing and recovery results in 5% BSA. (n = 3).

|  | Conc. | Concentration(x±S.D.) | | | | |
| --- | --- | --- | --- | --- | --- | --- |
| Cpd. | (ng/ml) | Autosampler | Bench-top 4 ºc | Bench-top RT | Freeze-thaw | Long-term |
| HCY | 40 | 99.67±12.81 | 97.09±3.76 | 105.34±4.74 | 94.25±6.56 | 102.82±2.91 |
|  | 200 | 93.90±6.53 | 101.78±7.23 | 100.10±7.41 | 107.49±6.49 | 101.23±2.23 |
|  | 1000 | 101.31±1.27 | 94.50±5.22 | 91.70±1.81 | 95.44±10.69 | 94.93±1.53 |
| GSH | 40 | 99.45±8.57 | 94.84±2.80 | 98.33±7.13 | 99.38±6.67 | 95.44±7.73 |
|  | 200 | 104.15±7.63 | 97.18±8.24 | 99.62±6.12 | 102.11±3.11 | 95.68±3.94 |
|  | 1000 | 96.94±8.76 | 93.26±3.94 | 101.09±6.53 | 101.43±5.73 | 100.57±7.06 |

# Supplementary Figures

# 2.1 Figure legends of supplementary material


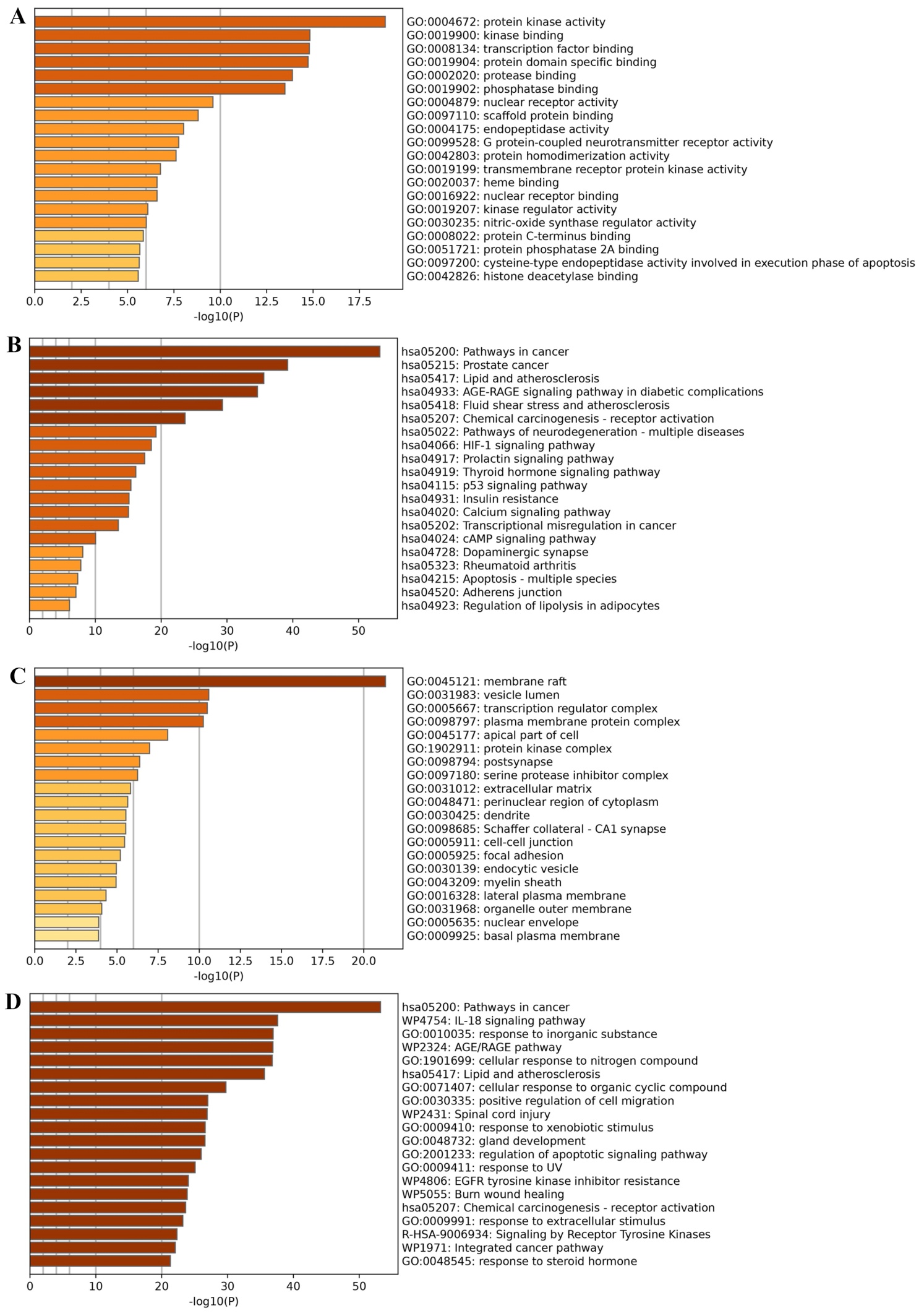


**Figure S1** Enrichment analysis of potential targets of honeysuckle-forsythia in the treatment of liver fibrosis.

(**A**) GO-molecular function analysis. (**B**) Kyoto Encyclopedia of Genes and Genomes (KEGG) analysis. (**C**) Gene Ontology (GO)-cell composition analysis. (**D**) GO-biological process analysis.


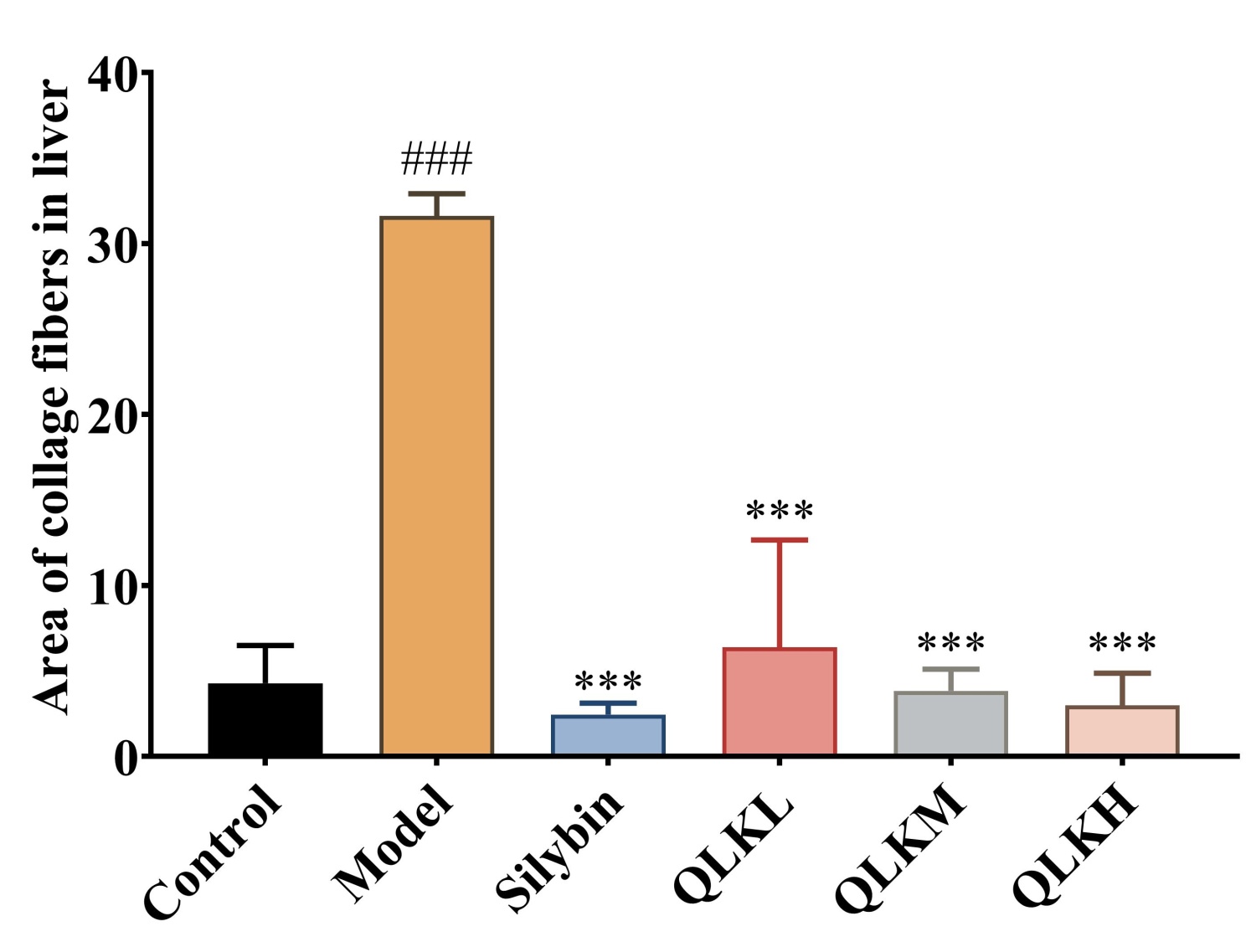


**Figure S2**  Area of collage fibers in liver according to masson stain. *** *P* < 0.005 vs. model, ^###^ *P* < 0.005 vs. control. QLK, quercetin-luteolin-kaempferol.

**Figure S3** Western blot analysis for TGF-1β and COX2 protein levels in the liver of model rats. (S1-1). GAPDH. left is GAPDH of COX2-1; middle is GAPDH of TGF-1β-1; right is GAPDH of TGF-1β-2. (S1-2) COX2. COX2-1 in **FIGURE 8A**. (S1-3) TGF-1β. left is TGF-1β-1; right is TGF-1β-2 in **FIGURE 8A**.

**Figure S4** Western blot analysis for COX2 protein levels in the liver of model rats. (S2-1). GAPDH. right is GAPDH of COX2-2. (S2-2). COX2. right is COX2-2.

**Figure S5** Western blot analysis for α-SMA protein levels in the liver of model rats. (S3-1). GAPDH. left is GAPDH of α-SMA-1in **FIGURE 8A**; middle is GAPDH of α-SMA-2**;** right is GAPDH of α-SMA-3. (S3-2).α-SMA. left is α-SMA-1; middle is α-SMA-2 in **FIGURE 8A;** right is α-SMA-3.

**Figure S6** Western blot analysis for TGF-1β and COX2 protein levels in the liver of model rats. (S4-1). GAPDH. left is GAPDH of TGF-1β-3; middle is GAPDH of COX2-3. (S4-2). TGF-1β-3. (S4-3). COX2-3.
