# Supplementary figures and images for: The combination of Lonicerae Japonicae Flos and Forsythiae Fructus herb-pair alleviated inflammation in liver fibrosis
Source: Front Pharmacol. 2022 Aug 19;13:984611. doi: 10.3389/fphar.2022.984611 (PMC9437263; doi:10.3389/fphar.2022.984611)

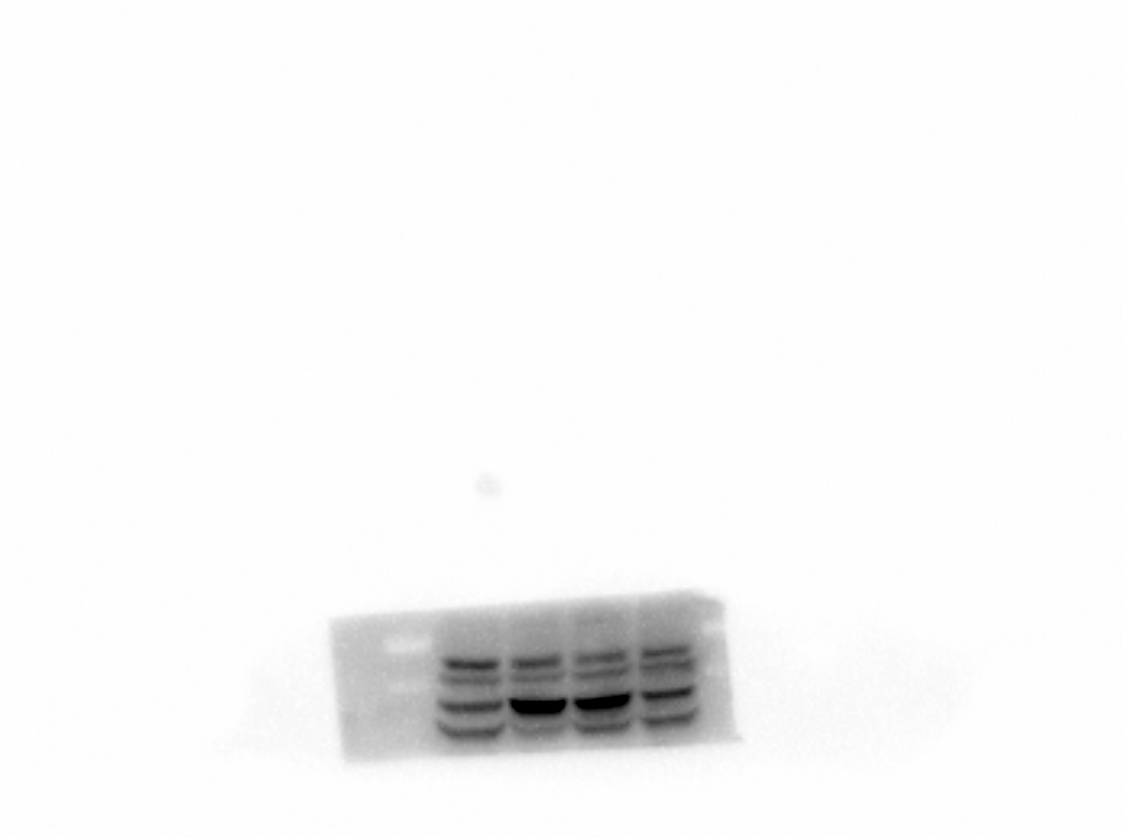

Supplement: Supplementary file 1 [file DataSheet1.zip › Supplementary material-The original results of Methodological evaluation and Western blot and figure legend--Revised version/FIgure 8 A/S1-2 COX2 in FIGURE 8A.tif]

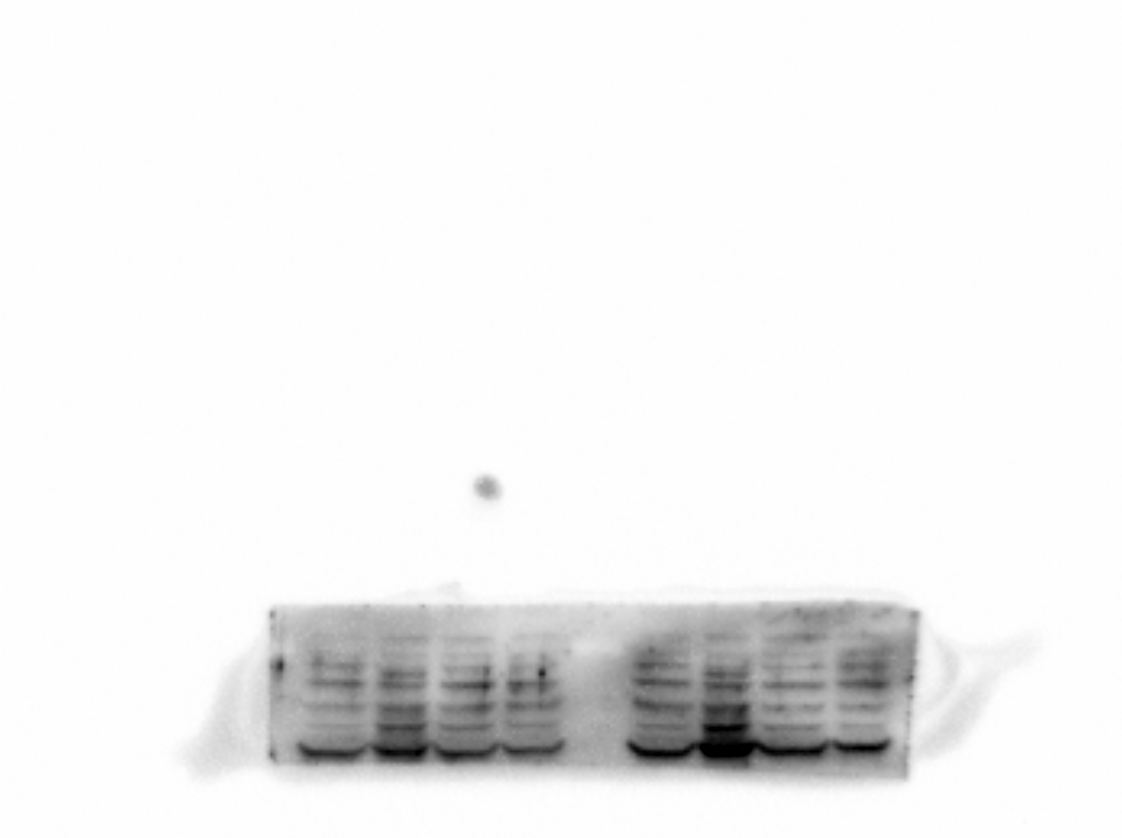

Supplement: Supplementary file 1 [file DataSheet1.zip › Supplementary material-The original results of Methodological evaluation and Western blot and figure legend--Revised version/FIgure 8 A/S1-3 TGF-1a┬--right in FIGURE 8A.tif]

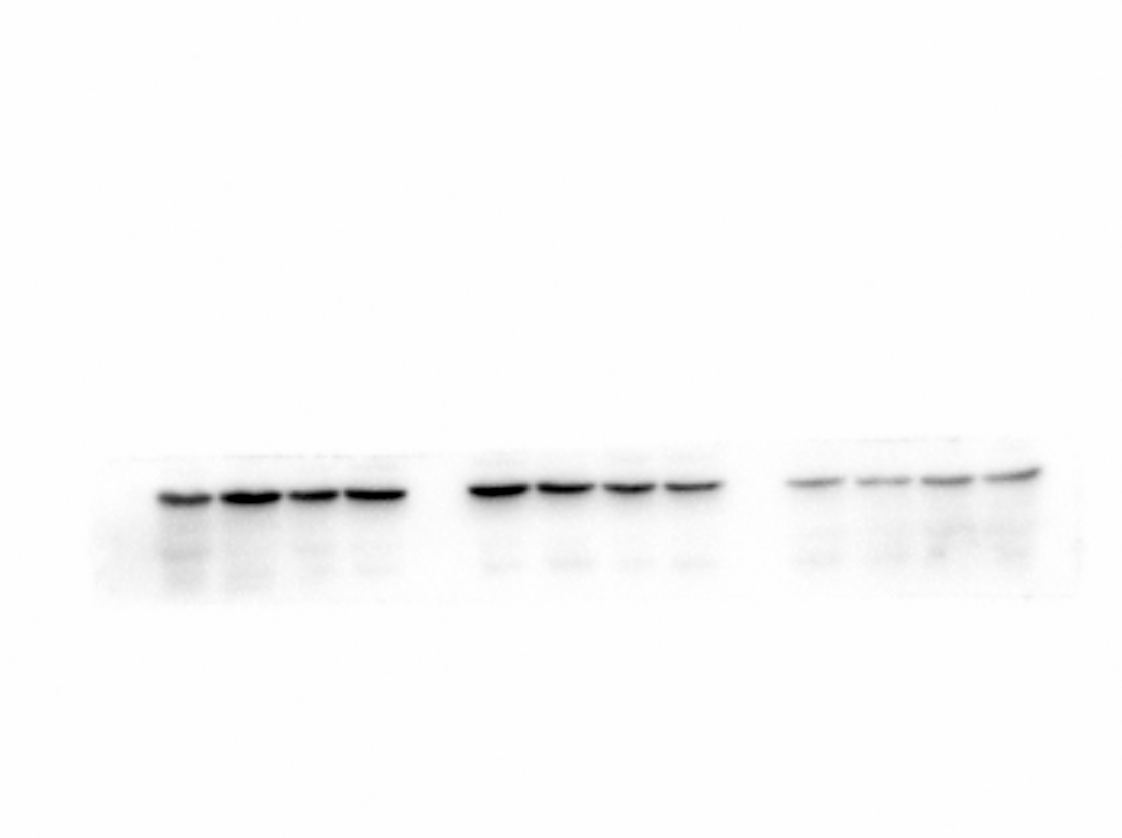

Supplement: Supplementary file 1 [file DataSheet1.zip › Supplementary material-The original results of Methodological evaluation and Western blot and figure legend--Revised version/FIgure 8 A/S3-1 GAPDH--left in FIGURE 8A.tif]

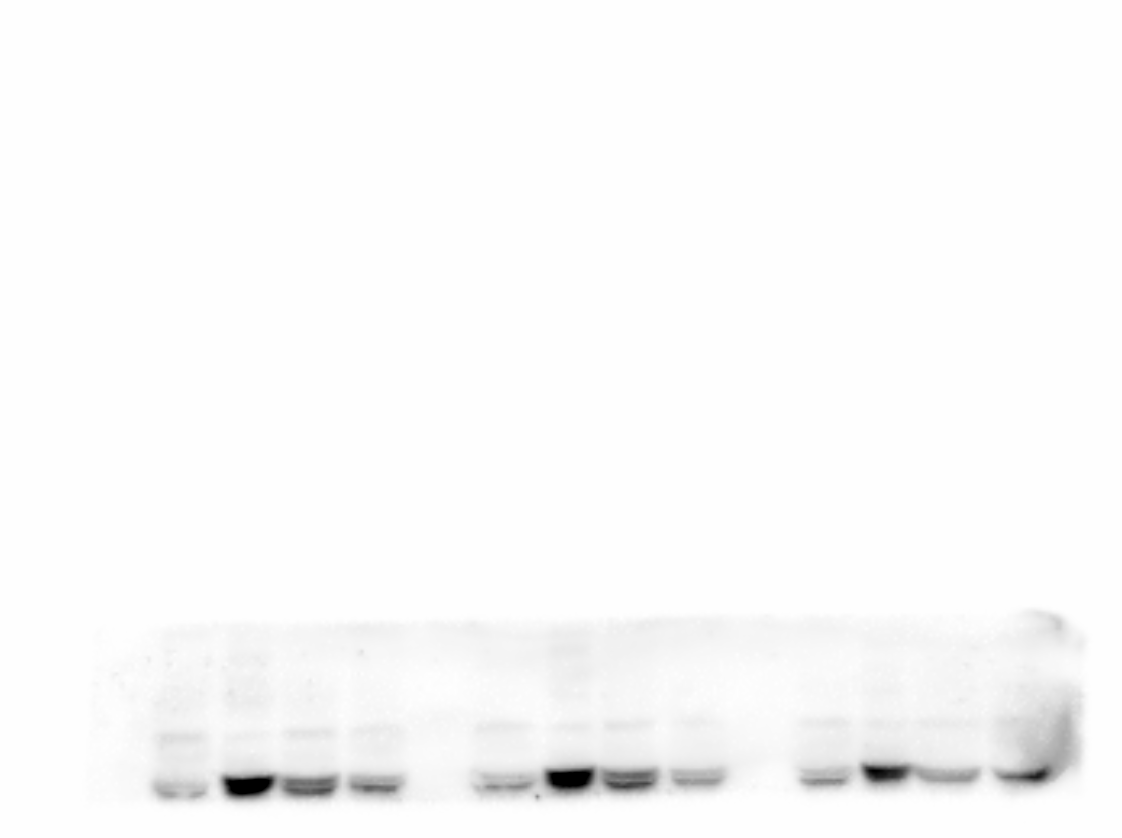

Supplement: Supplementary file 1 [file DataSheet1.zip › Supplementary material-The original results of Methodological evaluation and Western blot and figure legend--Revised version/FIgure 8 A/S3-2 a┴-SMA--middle in FIGURE 8A.tif]

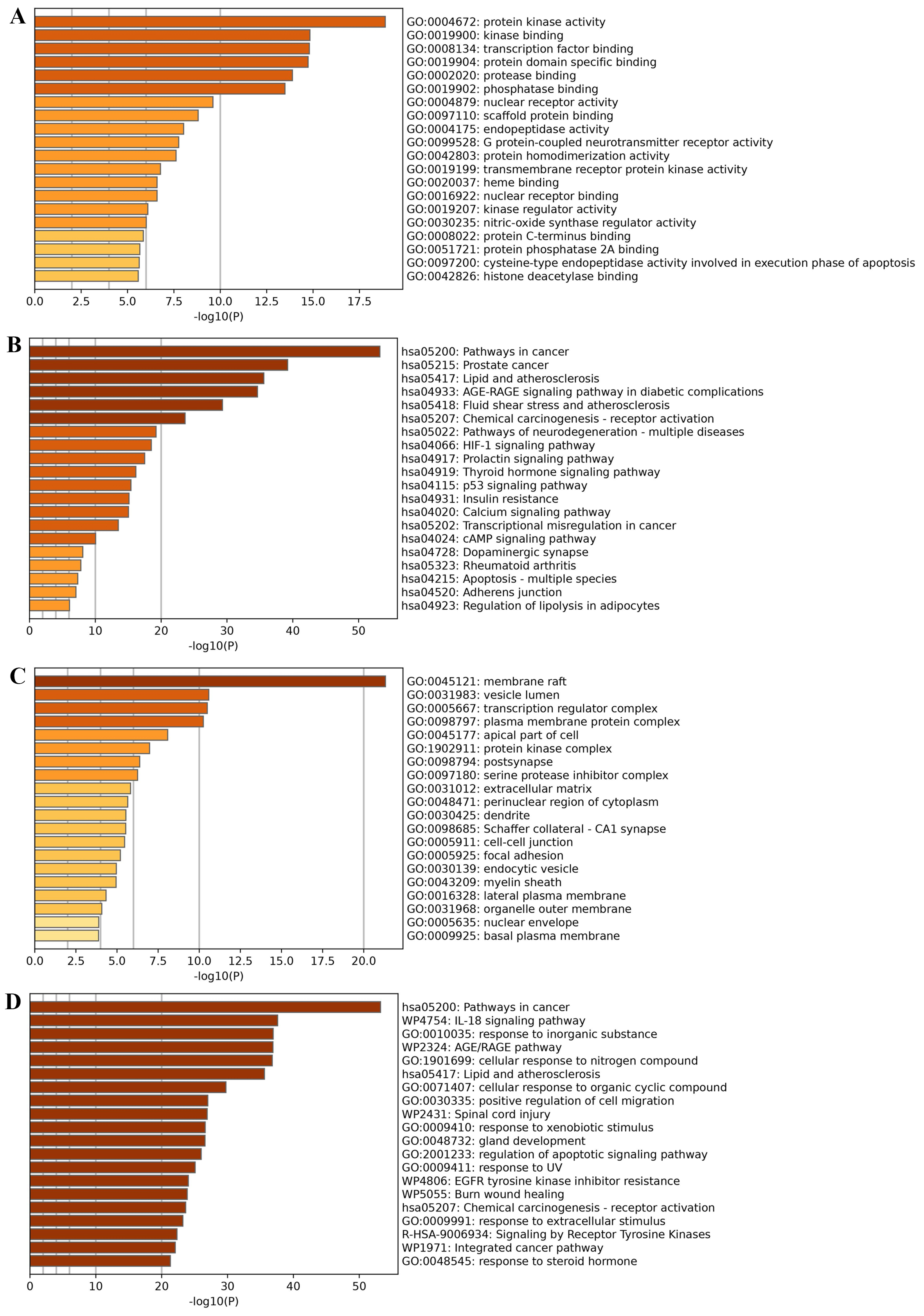

Supplement: Supplementary file 1 [file DataSheet1.zip › Supplementary material-The original results of Methodological evaluation and Western blot and figure legend--Revised version/Figure S1.jpg]

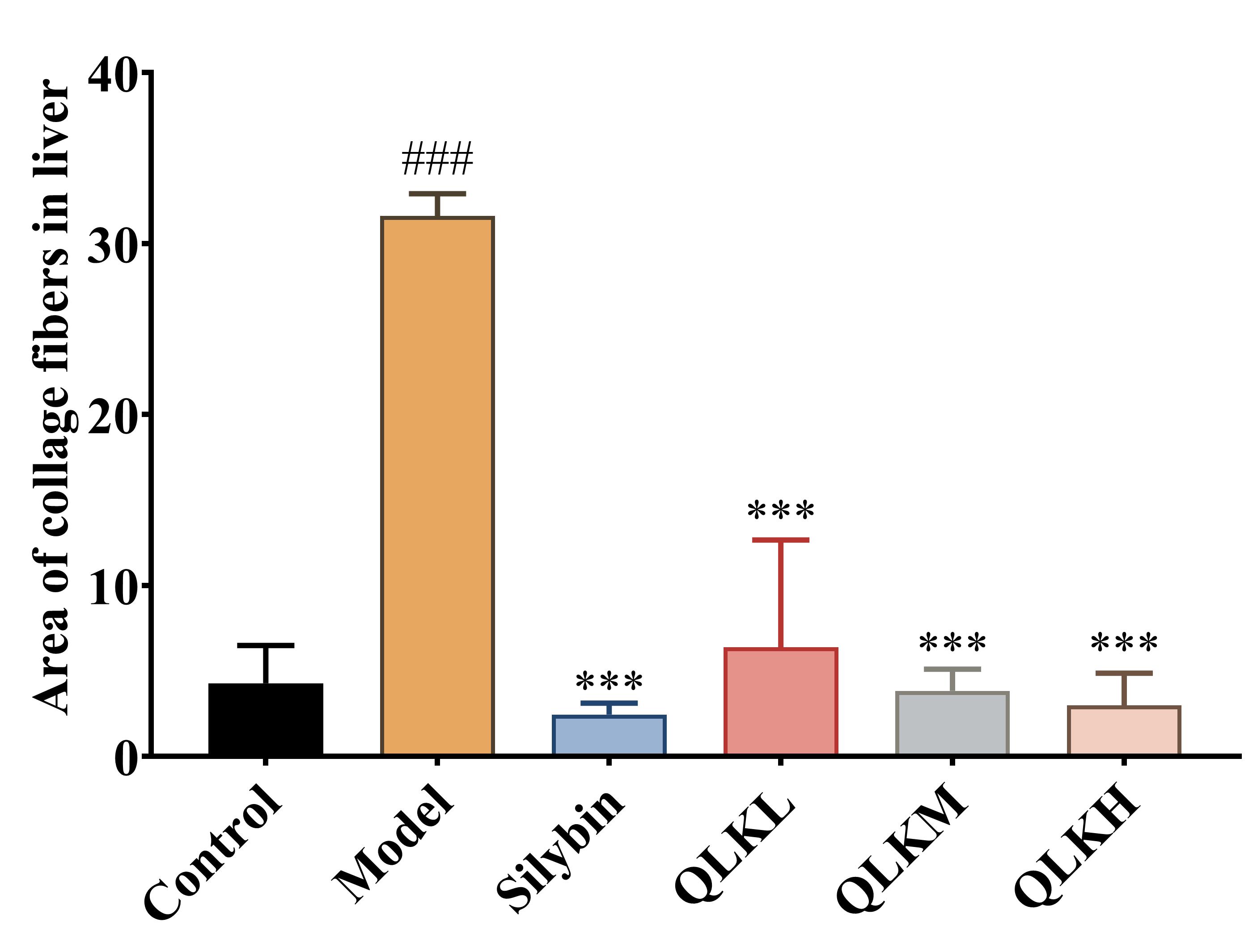

Supplement: Supplementary file 1 [file DataSheet1.zip › Supplementary material-The original results of Methodological evaluation and Western blot and figure legend--Revised version/Figure S2. Masson Stain.jpg]

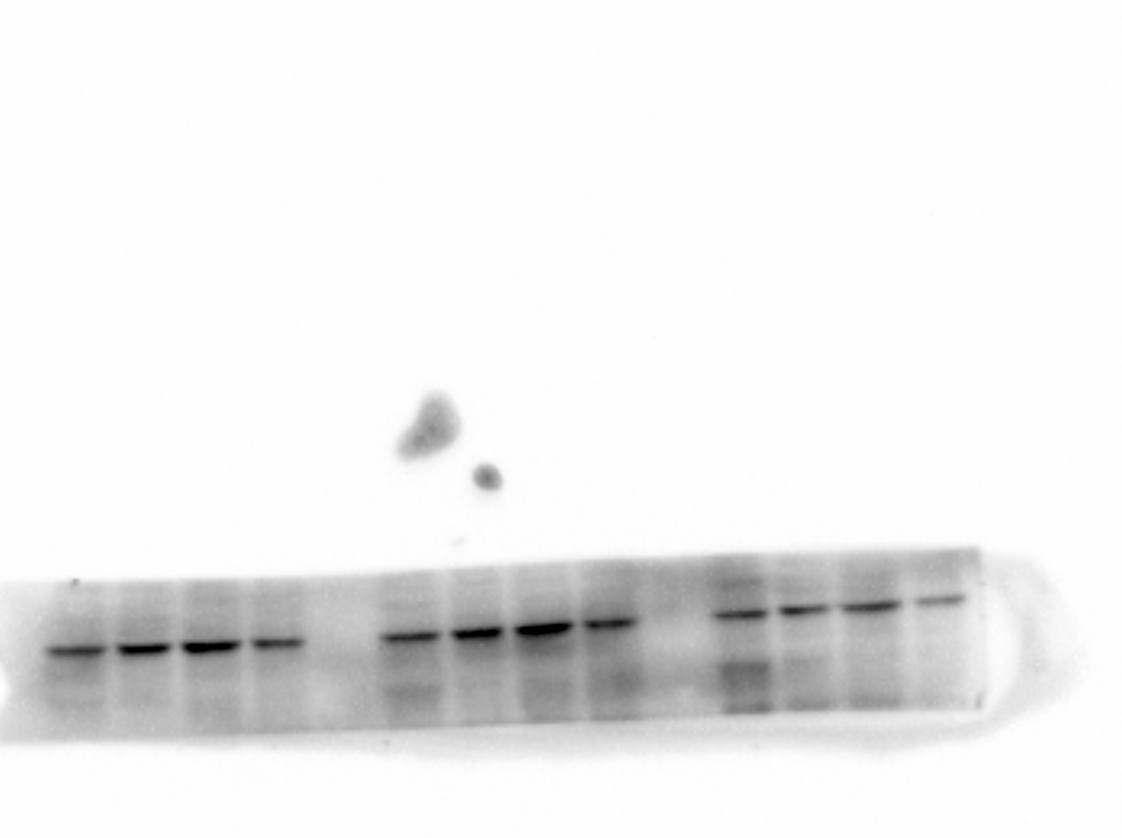

Supplement: Supplementary file 1 [file DataSheet1.zip › Supplementary material-The original results of Methodological evaluation and Western blot and figure legend--Revised version/Replication experiment/S1-1 GAPDH-left is GAPDH of COX2-1; middle is GAPDH of TGF-1a┬-1; right is GAPDH of TGF-1a┬-2.tif]

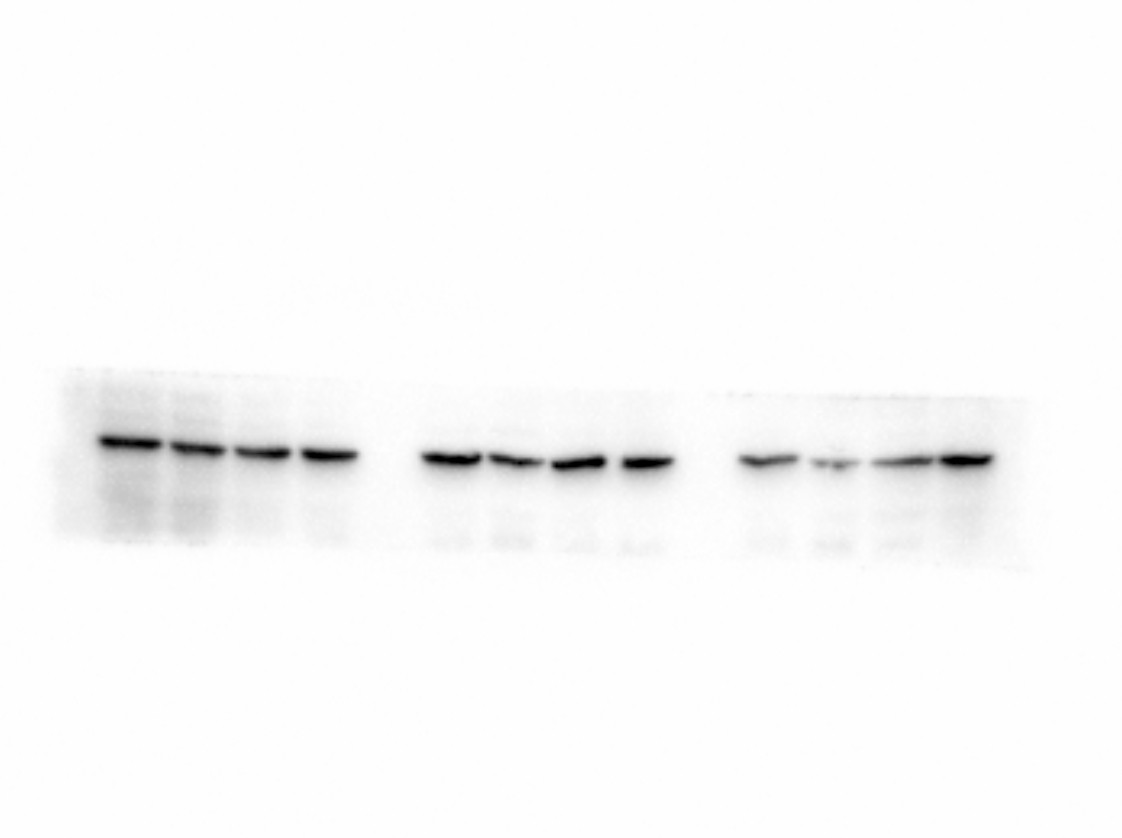

Supplement: Supplementary file 1 [file DataSheet1.zip › Supplementary material-The original results of Methodological evaluation and Western blot and figure legend--Revised version/Replication experiment/S2-1 GAPDH--right is GAPDH of COX2-2.tif]

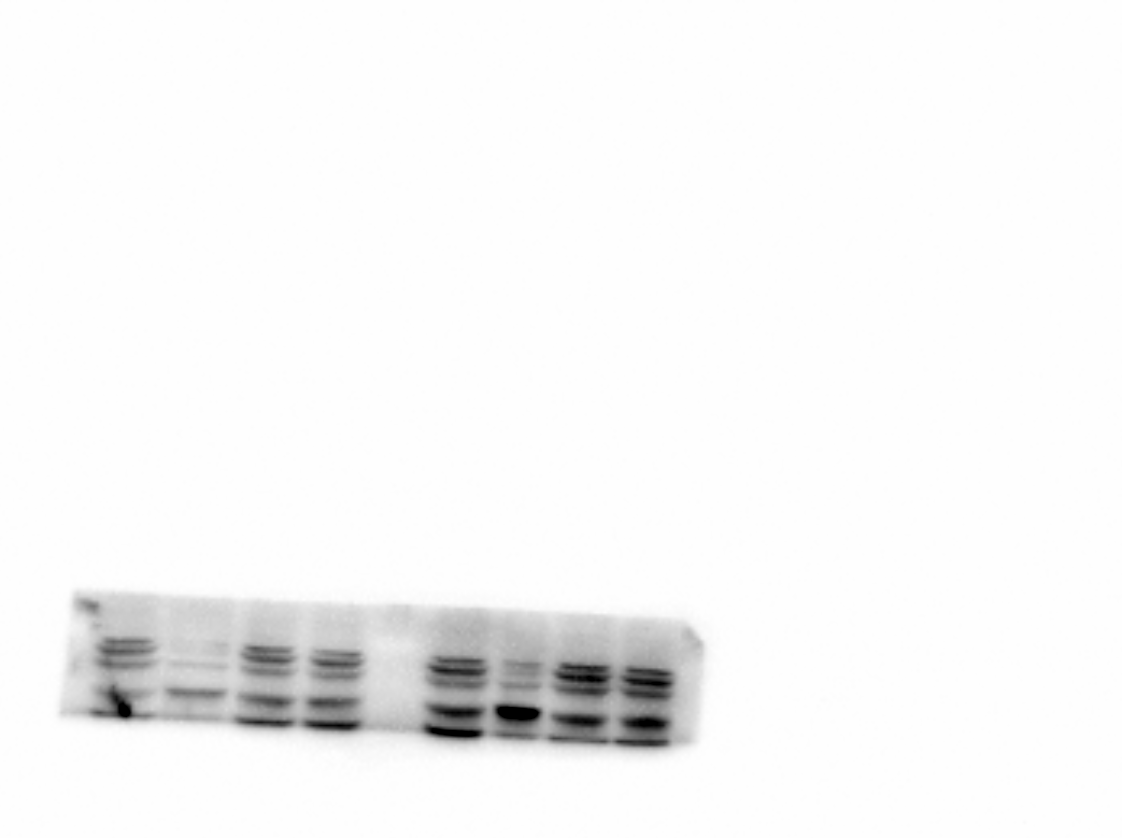

Supplement: Supplementary file 1 [file DataSheet1.zip › Supplementary material-The original results of Methodological evaluation and Western blot and figure legend--Revised version/Replication experiment/S2-2 COX2--right is COX2-2.tif]

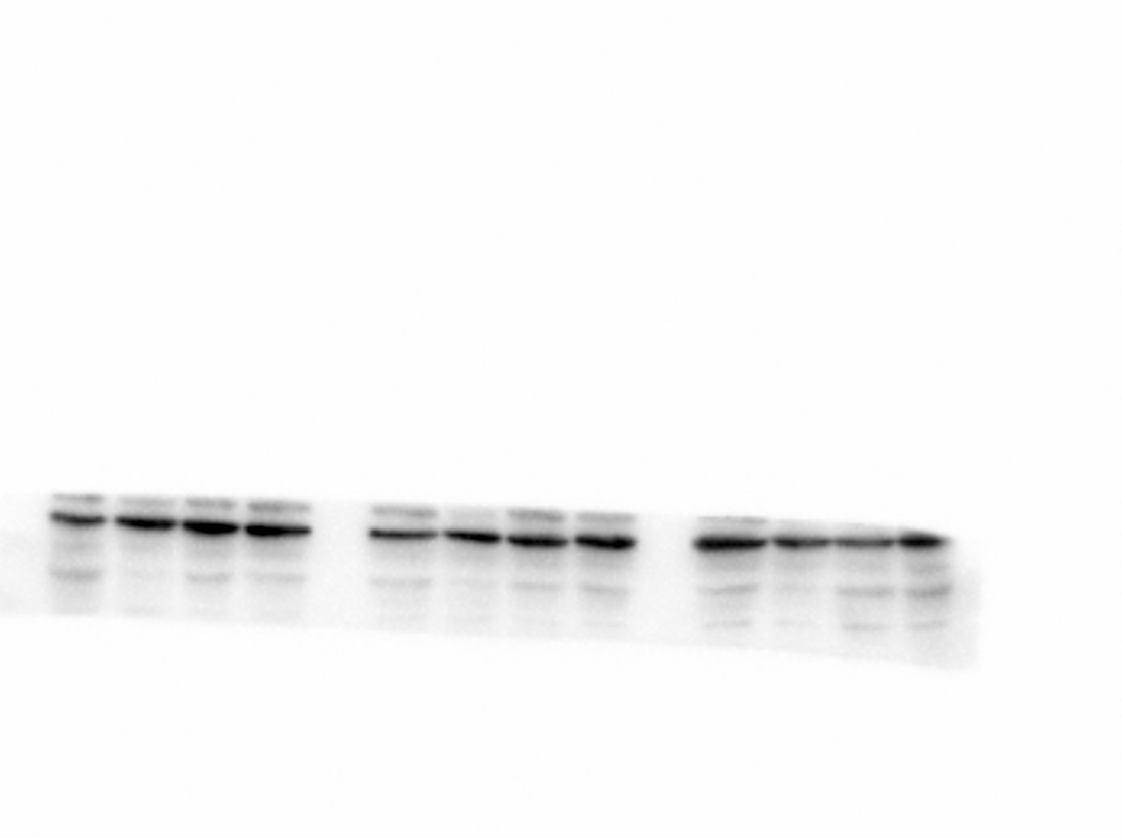

Supplement: Supplementary file 1 [file DataSheet1.zip › Supplementary material-The original results of Methodological evaluation and Western blot and figure legend--Revised version/Replication experiment/S4-1 GAPDH--left is GAPDH of TGF-1a┬-3; middle is GAPDH of COX2-3.tif]

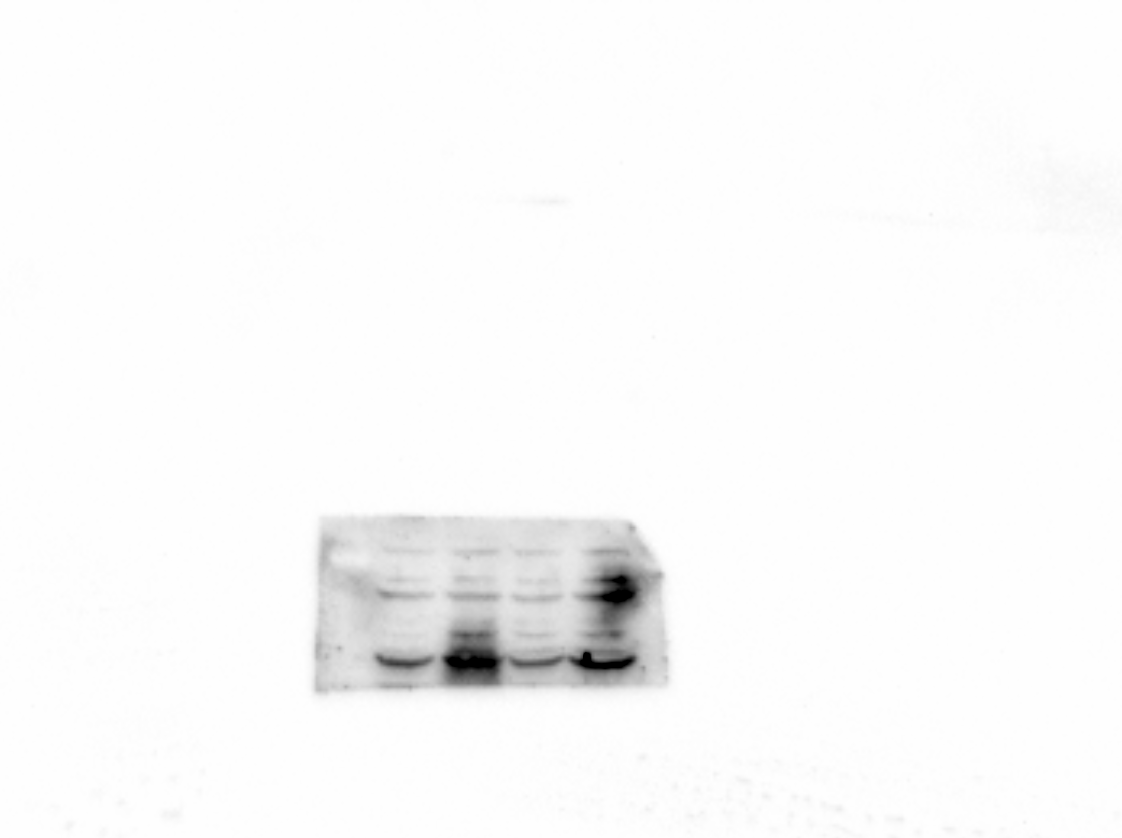

Supplement: Supplementary file 1 [file DataSheet1.zip › Supplementary material-The original results of Methodological evaluation and Western blot and figure legend--Revised version/Replication experiment/S4-2 TGF-1a┬-3.tif]

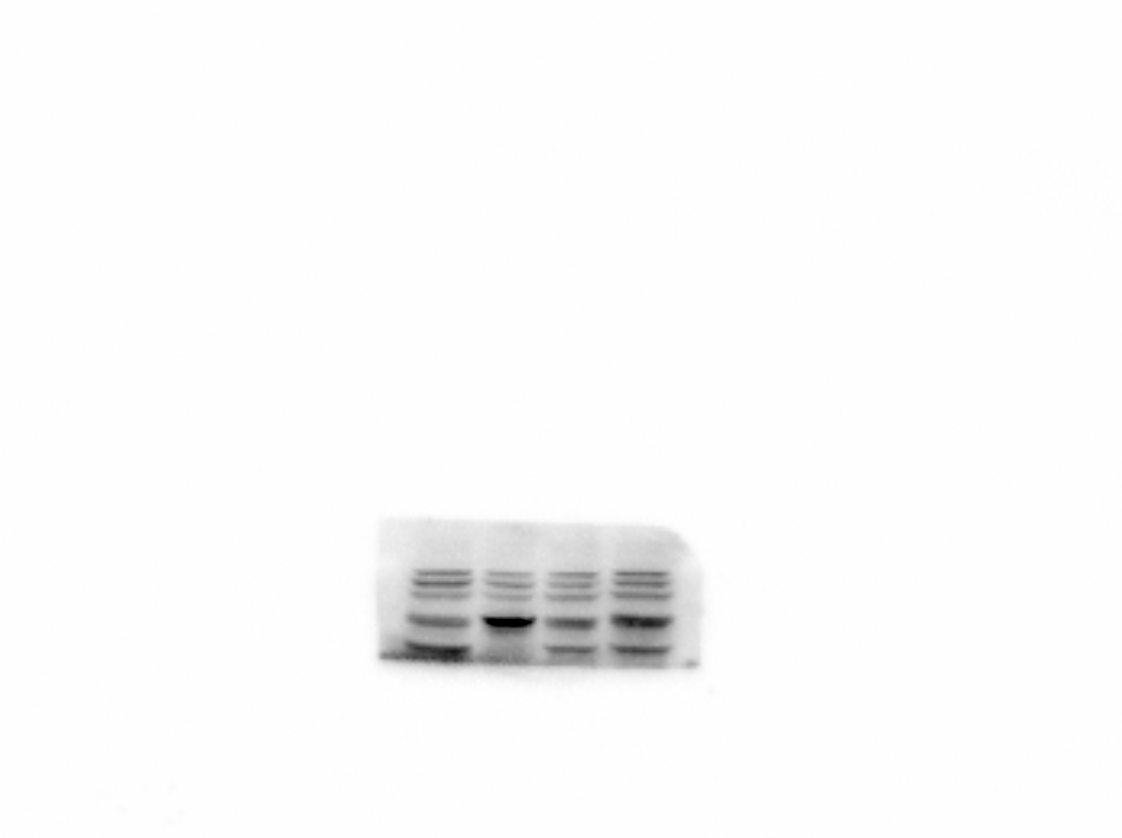

Supplement: Supplementary file 1 [file DataSheet1.zip › Supplementary material-The original results of Methodological evaluation and Western blot and figure legend--Revised version/Replication experiment/S4-3 COX2-3.tif]
